# Discovery of Muscle-Tendon Progenitor Subpopulation in Human Myotendinous Junction at Single-Cell Resolution

**DOI:** 10.34133/2022/9760390

**Published:** 2022-09-28

**Authors:** Ruojin Yan, Hong Zhang, Yuanzhu Ma, Ruifu Lin, Bo Zhou, Tao Zhang, Chunmei Fan, Yuxiang Zhang, Zetao Wang, Tianshun Fang, Zi Yin, Youzhi Cai, Hongwei Ouyang, Xiao Chen

**Affiliations:** ^1^Dr. Li Dak Sum & Yip Yio Chin Center for Stem Cells and Regenerative Medicine, and Department of Orthopedic Surgery of the Second Affiliated Hospital, Zhejiang University School of Medicine, Hangzhou, China; ^2^Department of Sports Medicine, Zhejiang University School of Medicine, Hangzhou, China; ^3^China Orthopedic Regenerative Medicine Group (CORMed), Hangzhou, China; ^4^Department of Orthopedic Surgery of Sir Run Run Shaw Hospital, Zhejiang University School of Medicine, Hangzhou, China; ^5^Zhejiang University-University of Edinburgh Institute, Zhejiang University School of Medicine, and Key Laboratory of Tissue Engineering and Regenerative Medicine of Zhejiang Province, Zhejiang University School of Medicine, Hangzhou, China; ^6^Department of Orthopaedic and Center for Sports Medicine, The First Affiliated Hospital, School of Medicine, Zhejiang University, Zhejiang, Hangzhou, China

## Abstract

The myotendinous junction (MTJ) is a complex and special anatomical area that connects muscles and tendons, and it is also the key to repairing tendons. Nevertheless, the anatomical structure and connection structure of MTJ, the cluster and distribution of cells, and which cells are involved in repairing the tissue are still unclear. Here, we analyzed the cell subtype distribution and function of human MTJ at single-cell level. We identified four main subtypes, including stem cell, muscle, tendon, and muscle-tendon progenitor cells (MTP). The MTP subpopulation, which remains the characteristics of stem cells and also expresses muscle and tendon marker genes simultaneously, may have the potential for bidirectional differentiation. We also found the muscle-tendon progenitor cells were distributed in the shape of a transparent goblet; muscle cells first connect to the MTP and then to the tendon. And after being transplanted in the MTJ injury model, MTP exhibited strong regenerative capability. Finally, we also demonstrated the importance of mTOR signaling for MTP maintenance by *in vitro* addition of rapamycin and *in vivo* validation using mTOR-ko mice. Our research conducted a comprehensive analysis of the heterogeneity of myotendinous junction, discovered a special cluster called MTP, provided new insights into the biological significance of myotendinous junction, and laid the foundation for future research on myotendinous junction regeneration and restoration.

## 1. Introduction

The myotendinous junction (MTJ) or called muscle-tendon junction is a complex and specialized area located at the interface between tendons and muscles with the main function of force transmission. Although the structure of muscles and tendons are various, they are closely related in space and function. Tendon is a highly organized connective tissue that can transmit force between muscle and bone [1]. The movement of bones is produced by the transmission of force from muscles to bones through tendons [2]. MTJ is a mixed tissue of muscles and tendons. At present, its cell and tissue structure are not clearly defined [3], and no one knows its specific shape [4]. We intend to uncover its mystery. Countless people around the world suffer from diseases related to muscles and tendons every year. Injuries at the junction of muscles and tendons are also common, and the recurrence rate of injuries is high, which is the main factor for permanent inability to continue physical activity. Therefore, it is important to study MTJ [5].

For tendon injuries, conservation treatment and surgery are common therapies [6]. However, this approach contributes to some problems. For example, the mechanical strength of the tissue after repair cannot reach the level before the injury. The repaired void is filled with disordered scar-like tissues [7]. In muscle injury, different degrees of muscle fiber necrosis will lead to the destruction of the continuity of muscle-muscle and muscle-muscle unit functions [8]. The regeneration of injured muscle fibers and the formation of stump scar tissue occur simultaneously. The two processes support and compete with each other. If there is more scar tissue, it may cause muscle fiber regeneration to stop [7, 9]. Therefore, if the regenerative capacity of cells is stronger than the formation of scar tissue, the damaged muscle can be repaired through regeneration methods. The MTJ is the place where muscles and tendons connect, and the above two kinds of injuries are prone to occur.

In recent years, the research of regenerative medicine has attracted more and more attention. The application of stem cell transplantation and tissue regeneration methods in the treatment of various diseases has increased rapidly [10]. Stem cell therapies for MTJ injuries are also a promising strategy, which requires us to understand the detailed structure and the most critical cell subsets that affect cell regeneration at the MTJ.

Scott et al. used *Hic1* to define resting mesenchymal progenitor subgroups with different functions and fates during skeletal muscle regeneration. They believed that *Hic1* regulates the quiescence of mesenchymal progenitor subgroups, and *Hic1+* progenitor cells can promote the regeneration of MTJ after trauma. But their function and fate in the process are still unclear [11]. Yaseen et al. found connecting cells with dual characteristics from MTJ and explored the underlying mechanism of musculoskeletal system construction focusing on MTJ [12]. However, the research was based on a mouse model, and there is still a certain gap with human MTJ mechanism research.

The microstructure of cells and extracellular matrix at the human's MTJ is still unclear. And the key cell subtype to regeneration is also remained unknown. The correct assembly of MTJ is essential for the correct understanding of muscle and tendon function. However, the existing research has not yet elucidated the signals that mediate the connection between muscle cluster and tendon cluster and the mechanisms that control the mutual induction of shared connection sites. The developmental pathways of muscle cells and tendon cells are still unclear, and the composition of specific cell clusters on MTJ is still controversial.

The single-cell sequencing technology that sequences the genome at the single-cell level can help us clarify these problems. It is suitable for analyzing the heterogeneity between cells and can reveal meaningful intercell gene expression variability [13].

In this article, we used single-cell RNA sequencing (scRNA-seq) to draw a comprehensive census map of all clusters on the MTJ. We have identified various cell clusters and their genetic markers to determine their differentiation relationships and characterize the diversity within specific cell types. We found key subgroups for muscle and tendon regeneration and obtained differentiation trajectories based on pseudotime. Then simulate and determine their differentiation relationship and the trajectory of their regulatory networks to find a cluster with common expression of muscle and tendon marker genes, which may be muscle-tendon progenitor cells (MTP). Then, we verified the existence of MTP cluster by fluorescent staining and hematoxylin-eosin (H&E) staining and observed the specific morphology of this cell subtype. Finally, we thought about the 3D structure of the MTP cluster. Our research showed the strategy of systematic transcriptome analysis of MTJ and discovered a group of special stem progenitor cells at the muscle-tendon junction and provided a blueprint for the use of regenerative methods to treat MTJ.

## 2. Results

### 2.1. scRNA-seq Analysis of the Cells on MTJ

**Figure 1:**
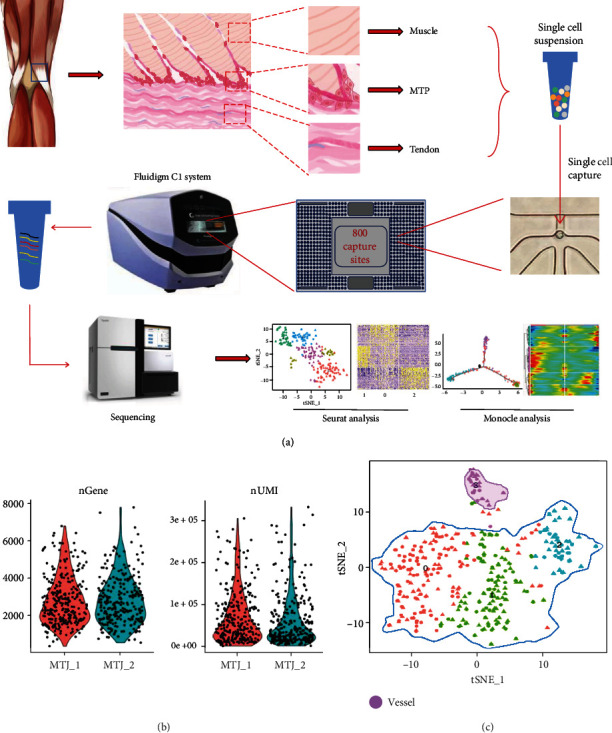
Overview of scRNA-seq analysis on muscle-tendon junction (MTJ) cells. (a) Process flow diagram of scRNA-seq analysis on MTJ. Single-cell samples of muscle cells, tendon cells were prepared for sequencing. Data analysis was performed using Seurat and Monocle. (b) Violin plots show the distribution of transcripts and genes detected per cell (patient MTJ_1 and patient MTJ_2). (c) t-SNE plot of single-cell samples profiled. Vessel cluster (purple circle); muscle-tendon junction clusters (blue circle). MTJ: muscle-tendon junction; MTP: muscle-tendon progenitor.

To systematically map the differentiation pathways of various cell clusters on MTJ, we prepared MTJ cells as single-cell samples (Figure 1(a)). After high-throughput single-cell sequencing and basic data processing, we obtained high-quality transcriptome data from 372 single-cell samples, including 195 samples from patient 1 and 177 samples from patient 2. We used these data for the next analysis. From Figure 1(b), we could know that most single-cell samples can be mapped to about 2000 genes (Figure S1c), which showed that scRNA-seq data have a good reads depth. After roughly controlling the single-cell data, we used Seurat to perform principal component analysis (PCA) and t-distributed random neighbor embedding (t-SNE) analysis [14].

**Figure 2:**
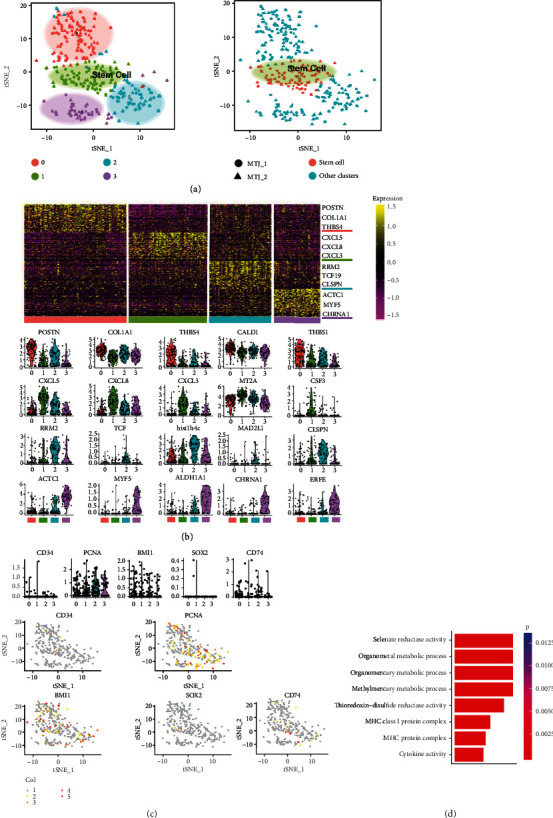
The identification and GO analysis of stem cells. (a) t-SNE plot of single-cell samples profiled. Stem cells cluster (green circle). (b) Violin plots show the expression level distributions of marker genes across clusters. Green indicates stem cell cluster. FeaturePlot of specific genes from stem cells subclusters. (c) FeaturePlot of specific genes from stem cell cluster. (d) GO analysis of stem cell cluster. GO: Gene Ontology; t-SNE: t-distributed random neighbor embedding.

First, we used the Seurat method to divide our sample into two main clusters, including a blood vessel cluster and a muscle and tendon mixed cluster (Figure 1(c)). The vascular cluster cells showed significant heterogeneity (Figure 1(c) and Figure S2a). After separating the vascular cluster cell population, we performed a follow-up analysis on the remaining muscle and tendon mixed cluster (Figure 2(a)).

We used the Seurat analysis method to perform cell cluster analysis on the muscle and tendon mixed cluster cells and obtained the t-SNE cell distribution map as shown in Figure 2(a). Observing the heatmap (Figure 2(b)), we found that there is a group of cells with high specificity, which is different from other cells except this group (group 1). Then, we drew a violin diagram of the first 5 highly expressed genes (*MT2A*, *CSF3*, *CXCL5*, *CXCL8*, and *CXCL3*) of this cell group (group 1) expressed on the whole muscle and tendon mixed cluster cells. The expression of these genes in other groups was much lower than that in this cell group (group 1), and FeaturePlot is also drawn to know the expression distribution of these genes on the mixed cluster of muscle and tendon. Next, we drew a violin diagram of the expression of stem cell marker genes (*CD34*, *PCNA*, *BMI1*, *SOX2*, and *CD74*) on the entire muscle and tendon mixed cluster cells and observed the expression of stem cell marker genes in these groups (Figure 2(c)). We found that the expression of these stem cell marker genes in group 1 was higher than that in other groups. Next, we performed Gene Ontology (GO) enrichment analysis on the main marker genes of group 1 and found that they have functions similar to those of stem cells (Figure 2(d)). Therefore, we defined this cell cluster (group 1) with stem cell characteristics as a stem cell cluster.

### 2.2. The Presence of MTP Cluster

**Figure 3:**
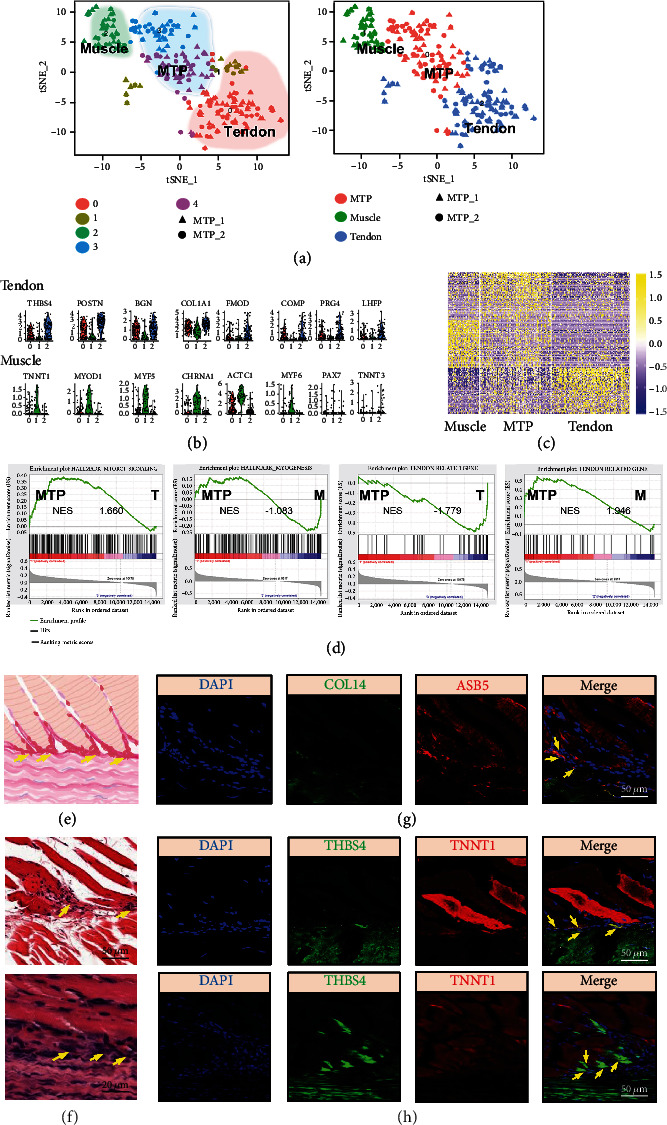
scRNA-seq analysis profiling reveals cell heterogeneity in tissues. (a) t-SNE plot of single-cell samples profiled. Muscle cluster (green circle), MTP cluster (blue circle), and tendon cluster (red circle). (b) Violin plots show the expression distributions of specific marker genes across sub-cluster. Clusters are represented by different colors. (c) Heatmap shows the expression pattern of top 50 differential genes in each cluster. Differential genes of each cluster are listed in Supplementary table. (d) The myogenesis signaling pathway and tendon-related gene in MTJ cluster compared with muscle or tendon cluster by GSEA signaling pathway analysis. (e) Schematic diagram of H&E staining for MTJ. Yellow arrows represent MTP. (f) H&E staining of muscle, tendon, and MTP in mouse MTP (top, scale bar = 50 *μ*m; bottom, scale bar = 20 *μ*m). Yellow arrows represent MTP. (g) Immunofluorescence staining for tendon cells (COL14, green) and muscle cells (ASB5, red) in human MTP; scale bar, 50 *μ*m. Yellow arrows represent MTP. (h) Immunofluorescence staining for tendon cells (THBS4, green) and muscle cells (TNNT1, red) in mouse MTP; scale bar, 50 *μ*m. Yellow arrows represent MTP.

According to the specific situation of differential gene expression, we divided the remaining cells (after removing the vascular cell cluster and stem cell cluster) into five clusters. Then, we integrated these five clusters into three clusters with different gene expression patterns based on the similarity and specificity of gene expression, including the subgroup with high expression of muscle markers (cluster 2) and the subgroup with high expression of tendon markers (clusters 0 and 1) and subgroups expressing both muscle and tendon markers (clusters 3 and 4) (Figures 3(a) and 3(b)).

Cluster 2 was associated with the high expression of muscle cell markers (such as *TNNT1*, *MYOD1*, *MYF5*, *CHRNA1*, *ACTC1*, *MYF6*, *PAX7*, and *TNNT3*), so this cluster was labeled as a muscle cluster (Figures 2(b) and 2(c)). Clusters 0 and 1 were labeled as tendon cluster that highly expressed*THBS4*, *POSTN*, *BGN*, *COL1A1*, *FMOD*, *COMP*, *PRG4*, and *LHFP* (Figures 3(b) and 3(c)). The expression patterns of cluster 3 and cluster 4 were similar, so we classified them into a subgroup, which expressed higher levels of muscle cell marker genes and tendon cell marker genes, but its expression level was lower than the subgroup to which these marker genes belong. For example, *THBS4* is a marker gene for tendon cells [15, 16]. This gene had the highest expression level in the subgroup labeled as tendon, followed by the second highest expression level in the subgroups labeled as cluster 3 and cluster 4, and finally, the lowest expression level in the subgroup labeled as muscle. The difference between the three was obvious. As for the muscle cell marker gene *ACTC1*, its expression level was just the opposite. The expression level of *ACTC1* was the highest in the muscle subgroup, followed by the subgroups of cluster 3 and cluster 4, and the tendon subgroup was the lowest. Therefore, we believed that this cluster might be a cluster of muscle and tendon progenitor cells and labeled it as the MTP cluster.

Subsequently, we performed GO enrichment analysis and established the GO term map of muscle, tendon, and MTP cluster (Figure S3) and also performed GSEA analysis (Gene Set Enrichment Analysis) on these clusters (Figure 3(d)). In this way, we couldknow the functions and pathways each cluster enriched to further validate our cell classification. For example, in terms of tendon-related genes, the tendon cluster was more enriched than the MTP cluster, and the MTP cluster was more enriched than the muscle cluster. This proved the correctness of our cluster definition from the perspective of gene enrichment (Figure 3(d)).

**Figure 4:**
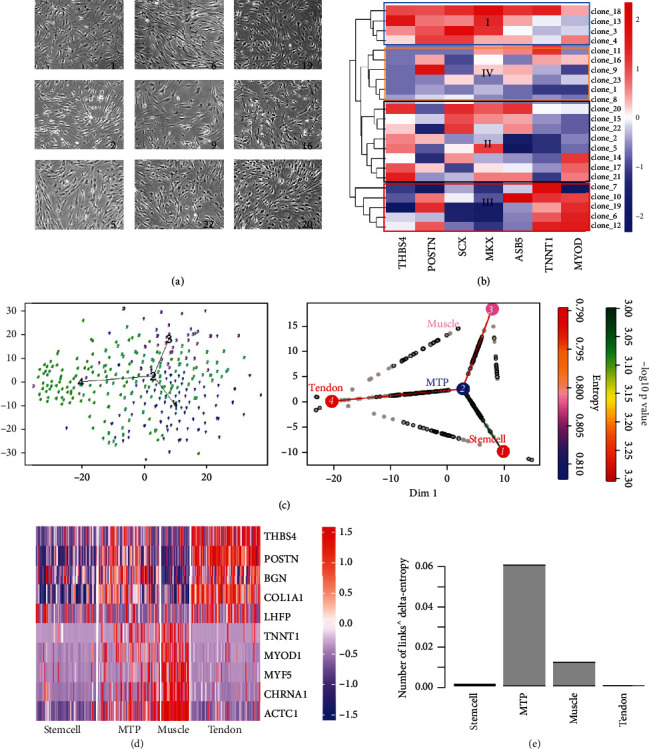
StemID identifies multifunctional cells in muscle-tendon junction cells. (a) Stem cell clones derived from MTP showed different morphology. (b) Heatmap of the expression levels of tendon and muscle marker genes for 23 clones. (c) Inferred hematopoietic lineage tree. Only significant links are shown (*p* < 0.01). The color of the link indicates the -log10 *p* value. The color of the vertices indicates the entropy. The thickness indicates the link score, reflecting how densely a link is covered with cells. (d) Heatmap shows the expression pattern of marker genes in each cluster. (e) Barplot of StemID scores for hematopoietic clusters.

According to the scoring bar chart of StemID, it showed that MTP cluster had the highest pluripotency (Figure 4(e)), indicating that MTP had the possibility of further differentiation and verifying the correctness of the definition of MTP. For stem cell cluster, its link score was high, but the entropy was small (Figure 4(c)). Among them, the link score represents the score of the possibility that the subgroup is connected with other subgroups, and the entropy value represents the possibility of the subgroup differentiated into other subgroups. Multiply the two to get the score of StemID. The higher the score, the higher the probability of differentiation into other subgroups. The entropy value of stem cell cluster wassmall, which led to a low score of StemID even if its link score was large. In combination with the results of the GO enrichment analysis we performed on this cluster (Figure 2(d)), we thought it might be a stem cell cluster in the dormant phase [17].

After clonal culture of MTJ-derived stem cells, we observed that different cloned cells showed different morphologies. (Figure 4(a)). Next, after drawing the heatmap of the expression levels of tendon and muscle marker genes for 23 clones, wefound that the heatmap was divided into 4 groups (Figure 4), one group (group 2) had high expression of tendon marker genes, another group (group 3) had highly expressed muscle genes, and the expression of tendon and muscle marker genes was highsimultaneously on one group (group 1), while the expression of the last group (group 4) cells was all low. This showed that there is a group of cells, between muscle and tendon. We believed that group 1 was MTP, group 2 was tendon, group 3 was muscle, and group 4 was a subgroup with low expression of muscle and tendon related genes. This proved the correctness of our grouping and the existence of MTP from another direction. Among them, clone 18 showed high expression of both tendon marker genes and muscle marker genes, which might be the MTP found by scRNA-seq.

### 2.3. Discovery of the Structure of MTP Cluster

Next, we performed H&E staining on the tissue sections, and found a circle of cells at the junction of muscle and tendon (Figure 3(f)). Previous studies suggested that the finger-like muscle cell membrane (sarcolemma) is directly connected to the collagen of the tendon [3]; however, through the results of H&E staining and the above-mentioned single-cell data analysis, it showed that, in fact, there is a subgroup of MTP with a ring structure in MTJ. In detail, the muscle cluster was first connected to this circle of MTP cluster and then connected to the tendon cluster. We have established a schematic diagram of the structure of MTJ (Figure 3(e)).

In order to further study the distribution of different clusters in tissues, we used dual-channel immunofluorescence staining to detect the expression of marker genes. The tendon cluster expressed THBS4, COL14 (green area), and the muscle cluster expressed ASB5, TNNT1 (red area). After integration, it was found that the MTP cluster (yellow area) co-expressing the muscle and tendon marker genes was located at the junction of the muscle and tendon, which proved the correctness of our conjecture, that was, the existence of the MTP cluster. Then, we discovered that the MTP cluster was a ring structure or, more accurately, a structure similar to a red wine glass.

### 2.4. Construction of MTJ Differentiation Trajectory

The in-depth study of single-cell gene expression enables researchers to describe complex biological processes or the transcriptional regulation process between highly heterogeneous cell populations. In the process of cell development, the state of the cell is constantly changing, so in many biological systems, the cell shows a continuous spectrum of states and may also involve the mutual conversion between different states. In fact, each cell is a snapshot of the transcription program being studied. The monocle package [18] can use advanced machine learning technology to use single-cell genome data to sort cells and construct cell lineage development, which is the pseudotime analysis method that allows us to understand the complex biology of cell development and differentiation. Pseudotime is an ordering of cells along the trajectory of a continuously developmental process in a system, which allows the identification of the cell types at the beginning, intermediate, and end states of the trajectory [19]. Therefore, we can use the monocle method to perform pseudotime analysis on scRNA-seq data to study the process of each cell state transition.

**Figure 5:**
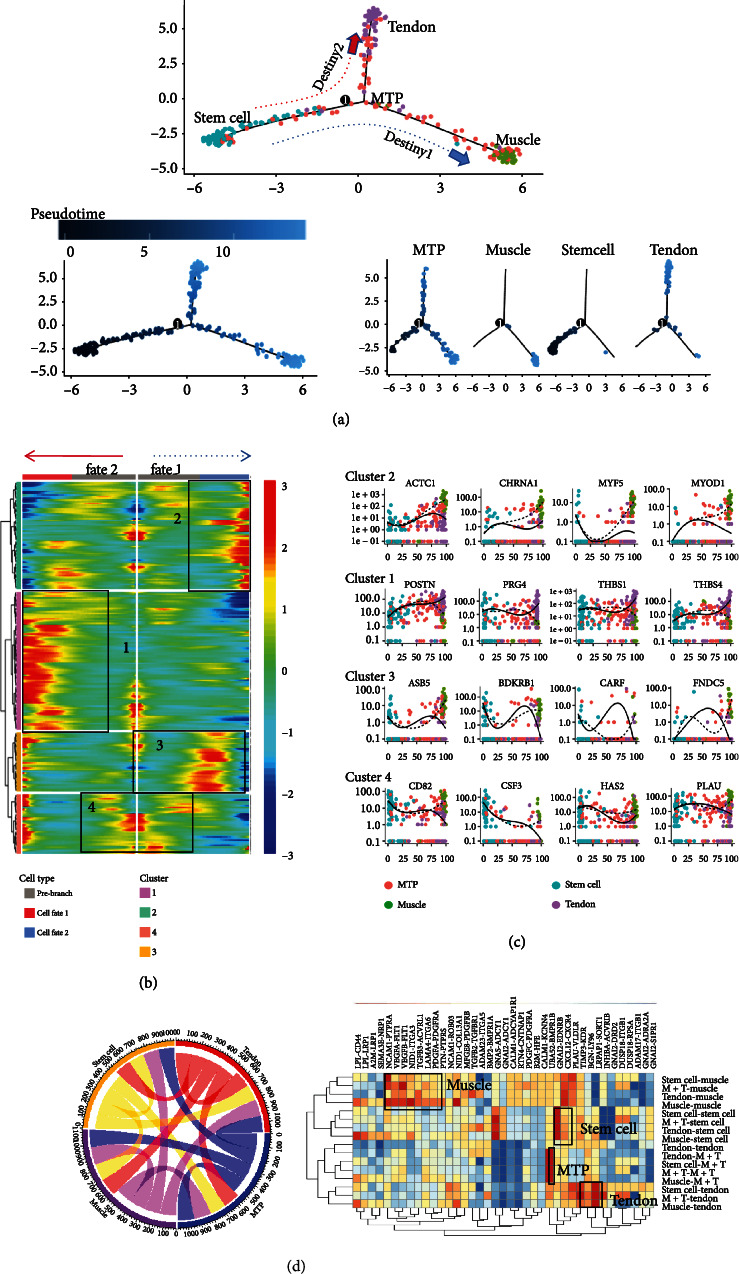
Pseudotemporal ordering of individual cells. (a) Differentiation trajectory of cells constructed by Monocle. Each data point represents a single cell colored by four clusters. (b) Heatmap shows the gene expression dynamics during cells profiled differentiation. (c) Genes (row) are clustered, and cells (column) are ordered according to the pseudotime development. Gene clusters 1-4 were selected for further analysis. (d) Connective diagram shows the interaction between muscle cluster, MTJ cell cluster, tendon cluster, and stem cell cluster.

We used the monocle2 package to sort the muscle, tendon, stem cell, and MTP cluster, and then we constructed a tree-shaped lineage differentiation trajectory (Figure 5(a)). The root of the differentiation trajectory is mainly composed of the stem cell cluster, while the turning point of the trajectory is mainly composed of the MTP cluster (Figure 5(a)). The two main ends of the trajectory were composed of the muscle cluster leading to Destiny 1 and the tendon cluster leading to Destiny 2 (Figure 5(a)). This differentiation trend was similar to the development process of differentiating muscles and tendons *in vivo*. A part of the stem cell population first differentiates into MTP cluster and then continueds to differentiate to obtain muscle cluster and tendon cluster, while other parts of stem cells differentiate directly into the muscle cluster or tendon cluster.

Since transcription factors (TFs) play a vital role in the regulation of development and differentiation, we used heatmap, which related to TFs, to show the gene expressions of these two branches (Figure 5(b)). On the basis of the gene expression observed from the heatmaps, we divided the TFs associated with each differentiation trajectory into four clusters (1, 2, 3, and 4) (Figure 5(b)). Then, we performed GO enrichment analysis on each cluster (Figure S4). We analyzed the genes that regulated the differentiation of muscles or tendons in the cell trajectories leading to different fates. From Figure 5(b), we could see that the TFs of cluster 4 (such as *CD82* and *CSF3*) had high expression levels on the stem cell population and then followed the pseudo-chronological changes to reach the MTP population and remain stable, and finally differentiated into two fates (Destiny 1 led to the muscle cluster, and Destiny 2 led to the tendon cluster). Then, we performed GO enrichment analysis on the four groups of TFs, and we knew that this group of TFs was closely related to cell mitosis (Figure S4).

The TFs of clusters 1 and 2 were highly expressed at the end of differentiation (Figure 5(c)). These TFs indicated the characteristics of each cell, including muscle cells (such as *ACTC1* [20, 21] and *CHRNA1* [22]) and tendon cells (such as *THBS4* and *POSTN*) [23]. *ACTC1* and *MYOD1*were slightly upregulated in the early differentiation process, significantly upregulated in the cells leading to Destiny 1, and downregulated in the cells leading to Destiny 2, which further proved that Destiny 1 led to muscle cluster and Destiny 2 led to tendon cluster (Figure 5(c)). These were likely to be consistent with the development of tendons and muscles in our body, which meant that the starting and ending points of our differentiation trajectory might be correct. The TFs of cluster 3 were highly expressed in the middle of differentiation. From the trajectory diagram, we knew that the arc top of the gene expression curve mainly included MTP (Figure 5(c)). These gene expression curves showed us the changes in gene expression of key TFs related to the differentiation process during the differentiation process and might provide evidence for the optimization of the *in vitro* differentiation system. Specifically, we constructed a differentiation trajectory (Figure S5) according to each cell type. Next, we mainly focused on the expression of tendon marker genes (*LHFP*, *POSTN* [24–26]) and muscle marker genes (*ACTC1*, *CHRNA1*) with obvious trends. In summary, the above results indicated that the differentiation trajectory of the entire MTP was likely to mimic the development *in vivo*.

### 2.5. Verification of the Bidifferentiation Capacity and Regenerative Function of MTP

**Figure 6:**
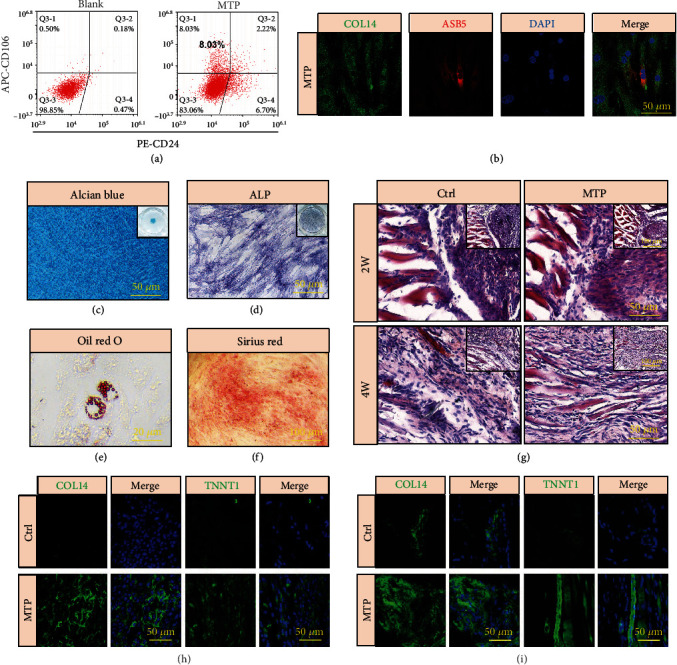
Characterization of the MTP cluster. (a) FACS analysis of CD106+CD24- MTP cluster in MTJ cells. (b) Immunofluorescence staining for COL14 of MTP cluster. Scale bars: 50 *μ*m. (c) Alcian Blue staining of MTP cluster. Scale bars: 50 *μ*m. (d) ALP staining of MTP cluster. Scale bars: 100 *μ*m. (e) Oil Red O staining of MTP cluster. Scale bars: 20 *μ*m. (f) Sirius Red staining of MTP cluster after tenogenic induction. Scale bars: 100 *μ*m. (g) Representative H&E images of the repaired MTJ in the Ctrl and MTP groups at two and four weeks after surgery. Scale bar: 50 *μ*m and 100 *μ*m. Immunofluorescence staining for COL14 and TNNT1 in the repaired MTJ tissues at two (h) and four (i) weeks after surgery. Scale bars: 50 *μ*m.

According to the marker gene, we found that MTP cluster was *CD106*+*CD24*- by single-cell analysis (Figure S6). Then, in order to investigate the function of MTP cluster, we separated CD106+CD24-MTP from MTJ cell populations by Fluorescence activated Cell Sorting (FACS), and found 8.03% MTJ cells were CD106+CD24- MTP (Figure 6(a)). To confirm that MTP cluster has the phenotype of both muscle and tendon, we applied immunofluorescence staining to prove that the MTP cluster both expressed tendon and muscle related makers (Figure 6(b) and S7), which indicated the MTP cluster has the potential to differentiate into muscle cells and tendon cells.

Next, we also characterized the multidifferentiation abilities of MTP cluster after lineage induction. Our results show that MTP could be induced toward chondrogenic, osteogenic, and adipogenic differentiation (Figures 6(c)–6(e)). After tenogenic induction, the MTP cluster also showed abundance collagen formation, as evidenced by Sirius Red staining (Figure 6(d)). Therefore, these results showed that the MTP cluster is multipotent, which were consistent with the results of scRNA-seq.

The efficacy of MTP cluster for MTJ regeneration *in vivo* was assessed in a nude mouse MTJ repair model (Figure S8a). The repaired tissues were evaluated at two and four weeks after surgery. H&E staining showed the MTP group had denser tissue than the Ctrl group both at two and four weeks (Figure 6(g)), and a more mature MTJ tissue morphology was exhibited in the MTP group at four weeks than that at two weeks. We also performed immunofluorescence staining to show that the expressions of COL14, THBS4, and TNNT1 were improved in the MTP group than the Ctrl group (Figures 6(h) and 6(i), S8b and S8c). Taken together, the repaired tissues from the MTP group exhibited better MTJ structures with enhanced expression of tendon and muscle related makers compared with those from the Ctrl group, demonstrating that MTP cluster has superior MTJ regeneration capacity.

### 2.6. Validation of the Importance of mTOR Signaling Pathway for MTP Function Maintenance

On the basis of the differentiation trend of MTP obtained from the above analysis, we could establish interaction diagrams between different cell types to gain a deeper understanding of the interactions between various groups of cells. From the expression of ligand or complementary receptor on each cell, we used the connection graph algorithm [27–29] to calculate the number of interactions between different cell types and showed the cell-cell interaction in the network graph (Figure 5(d)). The network diagram showed the connections between different cell types. The circle graph depicted the total number of ligand-receptor interactions between subclusters. The heatmap showed the highest receptor-ligand expression in the four clusters. From the heatmap, we could find that there is a one-to-many and many-to-one relationship between the receptor and the ligand. For example, the muscle ligand *FLT1* can bind to the receptor *VEGFA* on all types of cells, indicating the important role of muscle cells in the differentiation of other cell types. These ligand-receptor pairings may reveal cell-cell interactions during *in vivo* development.

We also used the scMLnet method [30] to analyze these four clusters, and obtained the interaction network diagram between the four clusters (Figure S11). By combining multilayer networks, this method is based on cell type specific gene expression, prior network information and statistics. Inferringly, a multilayer network is constructed by integrating the intercellular pathway (ligand-receptor interaction) and intracellular subnetwork (receptor-TF pathway and TF-target gene interaction). By connecting different microenvironmental cells to target cells, a multicellular network is constructed to clarify the microenvironment-mediated regulation of gene expression.

**Figure 7:**
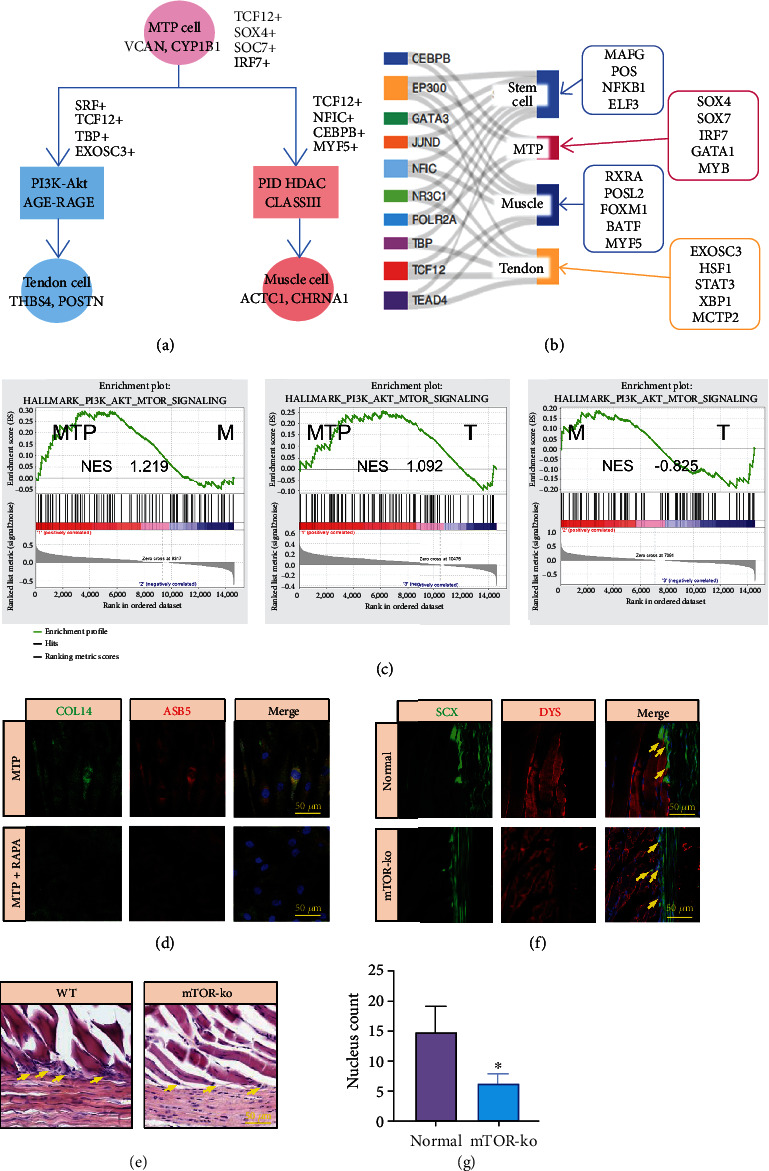
mTOR signaling pathway is vital for MTP maintenance. (a) Differentiation trajectories of MTP show key signaling pathways and TFs involved in the differentiation. (b) TFs that positively regulate key stem cell, MTP, muscle, and tendon markers. TFs on the left can regulate more than one marker, and marker-specific TFs are shown on the right. TFs and their targets are linked by lines. (c) GESA analysis: the function of mTOR signaling pathway in the differentiation process of clusters. (d) Immunofluorescence staining for COL14 and ASB5 of MTP and rapamycin treated MTP. Scale bars: 50 *μ*m. (e) H&E staining for WT and mTOR-ko mice. Scale bars: 50 *μ*m. Yellow arrows represent MTP. (f) Immunofluorescence staining for Scx and Dys of MTP in normal mice and in mTOR-ko mice. Scale bars: 50 *μ*m. Yellow arrows represent MTP. (g) Histogram of the number of nuclei in MTP (*p* < 0.05).

Summarizing the key signaling pathways and TFs of the four subgroups, we drew a snapshot of the scRNA-seq analysis of MTJ, showing the key signaling pathways and TFs involved in the differentiation of MTP cluster (Figure 7(a)). Then, Sankey diagram was used to show the active regulation of the key MTP, muscle, and tendon cluster marked by TFs.The TFs were predicted by the four subgroups of top 100 marker genes (Figure 7(b)).

We performed GSEA analysis on the muscle, tendon, stem cell, and MTP cluster, and we found that the mTOR signal pathway was significantly upregulated during the differentiation of MTJ cell population into muscle cluster, indicating that the mTOR signal pathway may be related to the differentiation of MTJ cells (Figure 7(c)).

To evaluate the role of mTOR signaling in the function maintenance of MTP cluster, we applied an mTOR inhibitor, rapamycin, to treat the MTP. We found that MTP lost their expression of tendon and muscle related makers after rapamycin treatment, as showed by immunofluorescence staining (Figure 7(d) and Figure S9), which proved that mTOR signaling might be vital for the phenotype maintenance of MTP cluster. We also used mTOR-ko mice for *in vivo* validation, as shown by H&E staining (Figure 7(e)), the number of MTP decreased in mTOR-ko mice. And immunofluorescence staining also showed that the number of MTP in the mTOR-ko group was significantly less than that in the normal group with reduced expression of Scleraxis (Scx) and Dystrophin (Dys) in mTOR-ko group (Figures 7(f) and 7(g)). Taken together, these results demonstrated the importance of mTOR signaling for the maintenance of MTP cluster.

In addition, we also showed the TFs that jointly regulates different cell types, as well as their functional enrichment network diagram, to understand the interconnection between the various groups of cells on the MTJ (Figure S10). Functionally, the MTP cluster showed high potential to differentiate into the muscle and tendon cluster lineages. These results provided valuable information for the optimization of the differentiation scheme. Network inferences from scRNA-seq data might reveal meaningful genetic correlations and provide biologically important insights.

## 3. Conclusion

MTJ is located at the interface between muscles and tendons. It is a complex and special area and the main part of force transmission. The structure of muscles and tendons is closely related in space and function. In this article, we used scRNA-seq data to group cells in the MTJ region. We found 4 different clusters, including tendon cluster, muscle cluster, MTP cluster, and stem cell cluster. We have identified many marker genes that could be compared with known cell types and locations. We found that there is a special subgroup in MTJ, which expresses both muscle marker genes (*TNNT1*, *MYOD1*, *MYF5*, *CHRNA1*, and *ACTC1*) and tendon marker genes (*THBS4*, *POSTN*, *BGN*, *COL1A1*, and *FMOD*). And through StemID analysis, we have known that this subgroup is more pluripotent than muscle and tendon cluster and has a certain differentiation potential, so we defined this subgroup as muscle-tendon progenitor cell subgroup. After drawing the heatmap of the qRT-PCR results from 23 cell clones, we observed the existence of cell clusters that also expressed muscle and tendon marker genes, which also proved the existence of MTP cluster from another perspective.

Using the monocle method, a tree-shaped lineage differentiation trajectory composed of four cell subgroups was constructed. The root of the differentiation trajectory was mainly composed of stem cell subgroups, and the turning point of the trajectory was mainly composed of MTP cluster. The two main ends of the trajectory were composed of muscle cluster leading to Destiny 1 and tendon cluster leading to Destiny 2. This differentiation trend was similar to the development process of differentiating muscles and tendons *in vivo*. Part of the stem cell population first differentiates into MTP cluster and then continues to differentiate to obtain muscle cluster and tendon cluster, while other stem cells directly differentiate into muscle cluster or tendon cluster.

Then, we observed H&E staining images and immunofluorescence staining images, which verified the presence of MTP cluster and also confirmed the structure of MTP, which is a transparent red cup-like structure.

We first isolated CD106+CD24-MTP from the MTJ cell cluster by FACS. We then applied immunofluorescence staining to confirm that MTP cluster both express tendon- and muscle-related markers, suggesting that MTP cluster has the potential to differentiate into muscle and tendon. We also proved MTP cluster has multi-differentiation capacity. And MTP also exhibited strong regenerative capability after transplanted in the MTJ injury model.

Using GSEA to analyze the signaling pathway, we found that the mTOR signaling pathway may be involved in the phenotype maintenance of MTP. And we also demonstrated the importance of mTOR signaling for MTP maintenance by *in vitro* addition of rapamycin and *in vivo* validation using mTOR-ko mice.

These results confirmed our hypothesis that MTP cluster is a group of cells located in the MTJ, shaped like a transparent wine glass, with the ability to differentiate into muscles and tendons. Our systematic analysis of the single-cell transcriptome provides new insights into the biological significance of MTJ and reveals the process of tendon muscle regeneration.

## 4. Discussion

In current study, we applied scRNA-seq analysis to detect the gene expression level of single cells in complex MTJ tissues and provided complete transcriptomics of MTJ. During the analysis of muscle and tendon related subgroups, we identified a new cell cluster, MTP cluster, which expresses muscle and tendon markers. In this study, scRNA-seq analysis of MTJ revealed continuous expression waves of major regulators of muscle tendon progenitor cell subsets that play a role in differentiation. The stratification and pseudotime sequencing determined the expression profile of MTP related to muscle and tendon development. The trajectory of the branches showed that there was a stem cell cluster in the resting phase in the early development of the muscle and tendon related populations, and the MTP cluster in the middle period; then a clear branch is formed, extending to the muscle cluster and tendon cluster. H&E staining and immunofluorescence staining show that MTP is in the shape of a red wine glass, connecting muscles and tendons. Our data will promote better understanding of the way muscles and tendons connect, the trajectory of muscle and tendon differentiation, and the composition of MTJ cell subsets at the cellular and molecular levels.

There is no definition of MTP in the current study. Most researchers believe that tendons and muscles in the complex tissue of the MTJ are differentiated from different progenitor cells [3], but there are also many researchers have also proposed different insights, such as Scott et al. [11] and Yaseen et al. [12]. Scott et al. suggested that *Hic1* regulates MP (mesenchymal progenitors) quiescence and identified a subset of MPs with transient and long-lasting roles in muscle regeneration [11]. We then drew a violin plot of *HIC1* on our single-cell data (Figure S9), and we found that *HIC1* was mainly expressed on the MTP cluster and stem cell cluster, which confirmed our results for the definition of single-cell clusters. Yaseen et al. identified a lateral plate mesoderm- (LPM-) derived fibroblast that could turn on the myogenic program to switch on myogenic characteristics at single-cell resolution, and found that LPM-derived fibroblasts could fuse into elongated muscle fibers by using live imaging and lineage tracing [12]. It was then proposed that the dual characterization of connective cells of the muscle-tendon in the mouse model might be a common mechanism for generating stable interactions between tissues throughout the musculoskeletal system. But the analysis was based on a mouse model and was not further explored using human tissues and cells; our study was based on single-cell data directly at the human muscle-tendon junction, which could directly reflect the cellular composition of human MTJs. Yaseen et al. proposed that *LoxL3* and *Prrx1* transcripts were predominantly expressed in fibrotic clusters but not myogenic clusters, and both genes were represented by dual-identity cell expression [12], but in our single-cell data, the expression of both *LOXL3* and *PRRX1* genes was roughly scattered among the various subgroups, and it is possible that differences in expression may be caused by species differences (Figure S12). Yaseen et al.'s article aroused our good thinking; the MTP cluster we found is likely to be formed by the fusion of fibroblast, or it may also be differentiated from stem cell cluster. However, the source of the MTP cluster needs to be further verified, and we may conduct in-depth research and discussion on its source in the future.

Currently, our research has found that MTP cluster, namely, muscle-tendon progenitor cell subgroup, has the ability to differentiate into muscles and tendons. From the perspective of gene expression, MTP expresses both muscle and tendon cell marker genes, and the expression levels are located in the middle of the respective subgroups of muscles and tendons, with a unique gene expression profile. The differentiation trajectory related to muscles and tendons indicated that the high expression of *PCNA* and *CXCL3* genes at the beginning of the trajectory could be defined as stem cells in the resting phase. The Destiny 1 branch presented a muscle differentiation pathway, and highly expresses muscle-lineage marker genes *ACTC1* and *MYF5*. Destiny 2 branch presented a tendon differentiation pathway and highly expresses tendon-lineage marker genes *THBS4* and *POSTN*. The middle inflection point and the transition of the branch trajectory was MTP, which inheritedfrom stem cells and extended to the muscles and tendons. From the perspective of cell pluripotency, the pluripotency of MTP is higher than that of muscle and tendon clusters. The cloned cells have high expression of muscle and tendon marker genes at the same time. They are a group of active progenitor cells that have the ability to differentiate into muscle and tendon. This discovery updates the current knowledge about the cell composition and cell differentiation of the tissue at the junction of muscles and tendons and opens a new door to the use of stem cells to regenerate and repair muscle and tendon injuries.

Muscle, which highly expresses *ACTC1* and *MYF5* genes, is located at the end of the developmental trajectory of muscle and tendon-related cells. It participates in various life-sustaining activities. Tendon, highly expressing *THBS4* and *POSTN* genes, is a highly organized connective tissue, similar to a rope, attaching itself to the bone, and then fixing one end of the muscle to the bone, which can transmit force between muscle and bone [1]. Without tendons, muscles cannot move. MTJ is a special anatomical area that connects muscles and tendons. The limited distribution of blood vessels and the relatively acellular characteristics directly lead to poor repair of tendon injuries. The inability of tendons to repair themselves and the overall inefficiency of current treatment options indicates that choosing an effective treatment strategy is the top priority. Repairing damaged MTJ by a regeneration method is worth studying. Our research has better analyzed the composition of muscle and tendon clusters at the cellular level and found the most critical MTP cluster in the process of muscle and tendon regeneration. This undoubtedly provides a new direction for the treatment of muscle and tendon.

As a special tissue connecting muscles and tendons, MTJ tissue plays a important role in the musculoskeletal system, so it is necessary to understand the way muscles and tendons connect. Previous studies on muscle-tendon connection models believe that muscles and tendons are directly connected in a finger-like manner [3]. In our research, H&E staining and immunofluorescence staining found that muscles are first connected to the MTP cell ring and then to the tendons, forming a structure similar to a wine glass filled with red wine. This discovery proved the existence of MTP cluster from the image aspect and also proves the position of MTP in the MTJ from the structural aspect. The muscles are surrounded by MTP, just like red wine in a red wine glass, and then connected to the tendons through the bottom of the MTP glass, so that two different tissues can also be closely connected to complete a series of physiological activities. Through single-cell analysis, we knew that the MTP cluster retains stem cell characteristics, also expresses both muscle and tendon marker genes, and may have the potential for bidirectional differentiation. And after being transplanted into the MTJ injury model, MTP also showed strong regenerative capacity. Therefore, it may be a potential seed cell for MTJ, tendon, or muscle injury repair.

## 5. Material and Methods

### 5.1. Human and Mouse Muscle-Tendon Junction Samples

All procedures and protocols for human samples were conducted with the informed consent and approval of the Ethics Committee of the Second Affiliated Hospital, School of Medicine, Zhejiang University (#16zju20160271). Human samples were used for cell culture, H&E staining, and immunofluorescence staining. All animal samples had ethical approval from the Institutional Animal Care and Use Committee of Zhejiang University (#zju20190049). Animal samples were used for H&E staining and immunofluorescence staining.

### 5.2. Isolation and Culture of Cells

Tissues were cut into pieces, digested with 0.2% type 1 collagenase, screened by low-density culture, and cultured in a basic medium [low-glucose Dulbecco's modified Eagle's medium (L-DMEM), 10% fetal bovine serum (FBS), and 1% penicillin-streptomycin] as described previously [31]. HTSPCs of the second to the fifth generation were used for further experiments. For rapamycin treatment, 2 nM rapamycin was added to the basic medium for 3 days.

### 5.3. Immunofluorescence Staining

We use paraffin sections for Immunofluorescence staining. See the supplementary for specific operations.

### 5.4. Histologic Evaluation

After being fixed in 4% (w/v) paraformaldehyde (PFA) for 24 hours, the samples were embedded in paraffin or optimal cutting temperature compound. Histologic sections (7 mm or 10 mm) for were prepared. As previously described [32], hematoxylin andeosin (H&E) staining was performed.

### 5.5. Assessment of Multi-Differentiation Capacity

The multi-differentiation potential of MTP cluster for osteogenesis, adipogenesis, chondrogenesis, and tenogenesis was investigated, as previously described [33].

### 5.6. Flow Cytometry Analysis and Sorting

MTJ cells were incubated with 1 *μ*g of APC-CD106 (Biolegend, 305810) and PE-CD24 (Biolegend, 311105) antibody for 30 minutes on ice, then washed with phosphate buffer saline (PBS), finally, the sample was analyzed and sorted with a flow cytometry tool (Beckman moflo Astrios EQ).

### 5.7. In *Vivo* Animal Experiments

To evaluate the MTJ formation ability of the MTP cluster, a nude mouse MTJ repair model was applied. All applicable institutional and/or national guidelines for the care and use of animals were followed. The study protocol was approved by the Zhejiang University Institutional Animal Care and Use Committee (#zju20190049). After anesthesia, we created a gap wound (0.1 mm in width and 0.3 mm in length) on each mouse's MTJ of the Achilles tendon as described previously [34]. 1 × 10^5^ MTP in 4 *μ*L fibrin gel were sutured to each patellar tendon defect in the MTP group, whereas in the control group (Ctrl), the MTJ defects were treated with 4 *μ*L of fibrin gel alone (Figure S8a). The wounds were then sutured. The rats were allowed free cage activity after surgery. At two and four weeks after cell transplantation, the repaired MTJs were assessed using H&E, and immunofluorescence staining. All experiments were performed using three independent samples from each group.

### 5.8. Single-Cell Capture, cDNA Library Preparation, and Sequencing

We use the Fluidigm C1 system and the C1 High Throughput Integrated Fluid Circuit (HT IFC) to perform single cell capture and library construction. See the supplementary for specific operations.

### 5.9. Processing of the scRNA-Seq Data

Raw sequencing reads was processed with Perl scripts to ensure the quality of data used in further analysis. For quality control, authors excluded cells in which less than 2000 genes or more than 8000 genes were detected and genes that are detected in less than 10 cells. After obtaining the digital gene expression data matrix, we used Seurat (2.4.3) for dimension reduction, clustering, and differential gene expression analysis in R (3.5.0). See the supplementary for specific process.

Then we used Metascape (http://metascape.org/) to perform Gene Ontology (GO) analysis on the respective Marker genes of each group, get the enrichment results, and compare which subgroups are mainly differentially expressed in functions. We ran StemID (https://github.com/dgrun/StemID) to get a histogram of differentiation potential scores. The digital gene expression matrix standardized by CPM (count-per-million) and the specific clustering information obtained after Seurat analysis is used as the input of monocle (monocle 2). Then, the marker genes of the four cell subgroups were used as differential genes, and monocle analysis was performed to obtain the differentiation trajectories of the four subgroups on the pseudo-time series. Connective map method was used to get the interaction between the four subgroups. GSEA was used for GO analysis and signaling pathway analysis. Taking the TFs predicted by the four groups of top100 marker genes on cytoscape (3.8.0) as input, draw a Sankey diagram to show the activity regulation of key stem cell, MTP, muscle, and tendon cluster labeled by TFs.

## Data Availability

The raw sequence data reported in this paper have been deposited in the Genome Sequence Archive (Genomics, Proteomics & Bioinformatics 2021) in National Genomics Data Center (Nucleic Acids Res 2022), China National Center for Bioinformation/Beijing Institute of Genomics, Chinese Academy of Sciences (GSA-Human: HRA002803), that are publicly accessible at https://ngdc.cncb.ac.cn/gsa-human. All data needed to evaluate the conclusions in the paper are available in the NGDC or present in the paper and/or the Supplementary Materials. Additional data related to this paper may be requested from the authors.

## Abbreviations

GO: Gene Ontology
GSEA: Gene Set Enrichment Analysis
MTJ: Muscle-tendon junction
MTP: Muscle-tendon progenitor
PCA: Principal component analysis
scRNA-seq: Single-cell RNA sequencing
TFs: Transcription factors
t-SNE: t-distributed random neighbor embedding.

## Supplementary Materials

Supplementary Materials Table: Differential genes of four clusters. Figure S1: quality control of the data set. Figure S2: the identification and gene ontology (GO) analysis of vessel cells. Figure S3: the GO terms corresponding to the three clusters. Figure S4: the GO terms corresponding to the four clusters. Figure S5: differentiation trajectories of cells. Figure S6: scRNA-seq analysis of CD106 and CD24 expression in MTJ. Figure S7: immunofluorescence staining for THBS4 of MTP. Figure S8: the efficacy of MTP cluster for MTJ regeneration in a nude mouse MTJ repair model. Figure S9: immunofluorescence staining for THBS4 of MTP and rapamycin treated MTP. Figure S10: use metascape to perform network enrichment analysis on the list of genes regulated by TFs shown in Figure 7(c). Figure S11: differentiation trajectories of cells. Figure S12: violin plots show the expression of HIC1, LOXL3, and PRRX1. Methods: method-related supplements.
