# Supplementary material for: Discovery of Muscle-Tendon Progenitor Subpopulation in Human Myotendinous Junction at Single-Cell Resolution
Source: Research (Wash D C). 2022 Sep 28;2022:9760390. doi: 10.34133/2022/9760390 (PMC9555880; doi:10.34133/2022/9760390)
Supplement: Supplementary Materials — Table: Differential genes of four clusters. Figure S1: quality control of the data set. Figure S2: the identification and gene ontology (GO) analysis of vessel cells. Figure S3: the GO terms corresponding to the three clusters. Figure S4: the GO terms corresponding to the four clusters. Figure S5: differentiation trajectories of cells. Figure S6: scRNA-seq analysis of CD106 and CD24 expression in MTJ. Figure S7: immunofluorescence staining for THBS4 of MTP. Figure S8: the efficacy of MTP cluster for MTJ regeneration in a nude mouse MTJ repair model. Figure S9: immunofluorescence staining for THBS4 of MTP and rapamycin treated MTP. Figure S10: use metascape to perform network enrichment analysis on the list of genes regulated by TFs shown in Figure 7(c). Figure S11: differentiation trajectories of cells. Figure S12: violin plots show the expression of HIC1, LOXL3, and PRRX1. Methods: method-related supplements. [file 9760390.f1.zip › Supplementary Material.docx]

**Supplementary Material**

Table: Differential genes of four clusters. Figure S1: Quality control of the data set. Figure S2: The identification and gene ontology (GO) analysis of vessel cells. Figure S3: The GO terms corresponding to the three clusters. Figure S4: The GO terms corresponding to the four clusters. Figure S5: Differentiation trajectories of cells. Figure S6: ScRNA-seq analysis of *CD106* and *CD24* expression in MTJ. Figure S7: Immunofluorescence staining for THBS4 of MTP. Figure S8: The efficacy of MTP cluster for MTJ regeneration in a nude mouse MTJ repair model. Figure S9: Immunofluorescence staining for THBS4 of MTP and rapamycin treated MTP. Figure S10: Use metascape to perform network enrichment analysis on the list of genes regulated by TFs shown in Figure 7c. Figure S11: Differentiation trajectories of cells. Figure S12: Violin plots show the expression of *HIC1, LOXL3* and *PRRX1*. Methods: Method-related supplements.

**Figures**

**
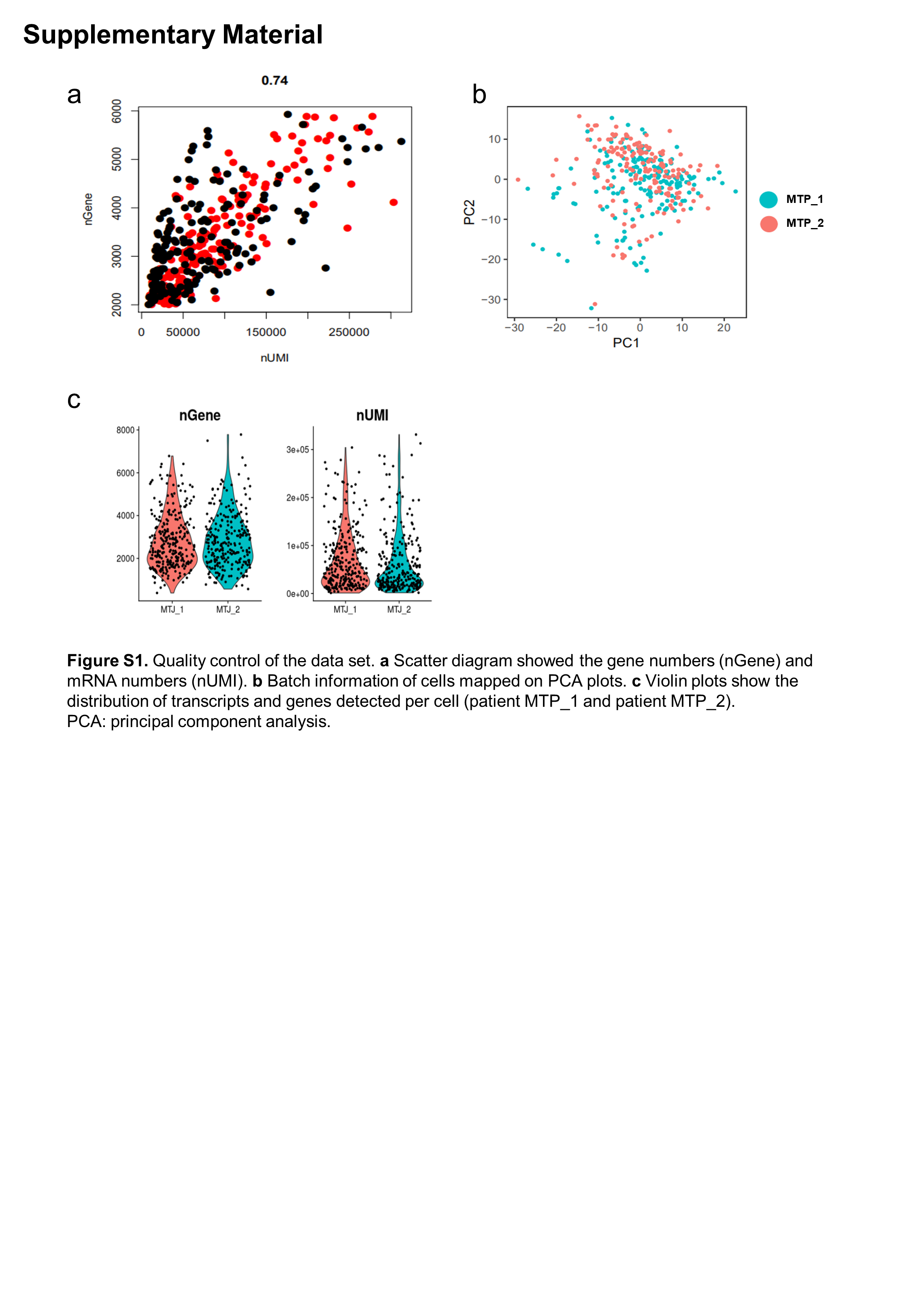
**

**Figure S1.** Quality control of the data set. **a** Scatter diagram showed the gene numbers (nGene) and mRNA numbers (nUMI). **b** Batch information of cells mapped on PCA plots. **c** Violin plots show the distribution of transcripts and genes detected per cell (patient MTP_1 and patient MTP_2). PCA: principal component analysis.


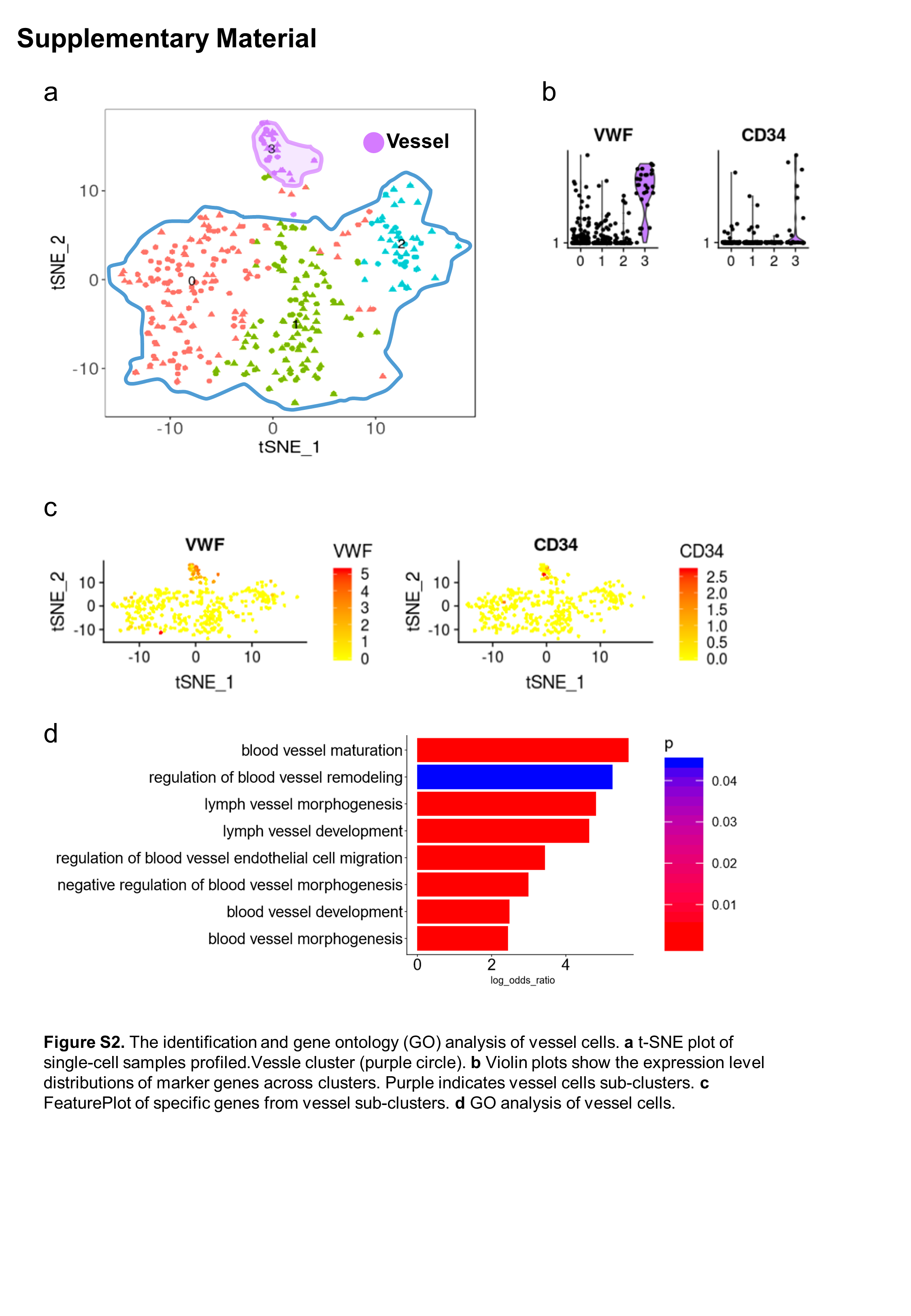


**Figure S2.** The identification and gene ontology (GO) analysis of vessel cells. **a** t-SNE plot of single-cell samples profiled.Vessle cluster (purple circle). **b** Violin plots show the expression level distributions of marker genes across clusters. Purple indicates vessel cells sub-clusters. **c** FeaturePlot of specific genes from vessel sub-clusters. **d** GO analysis of vessel cells.


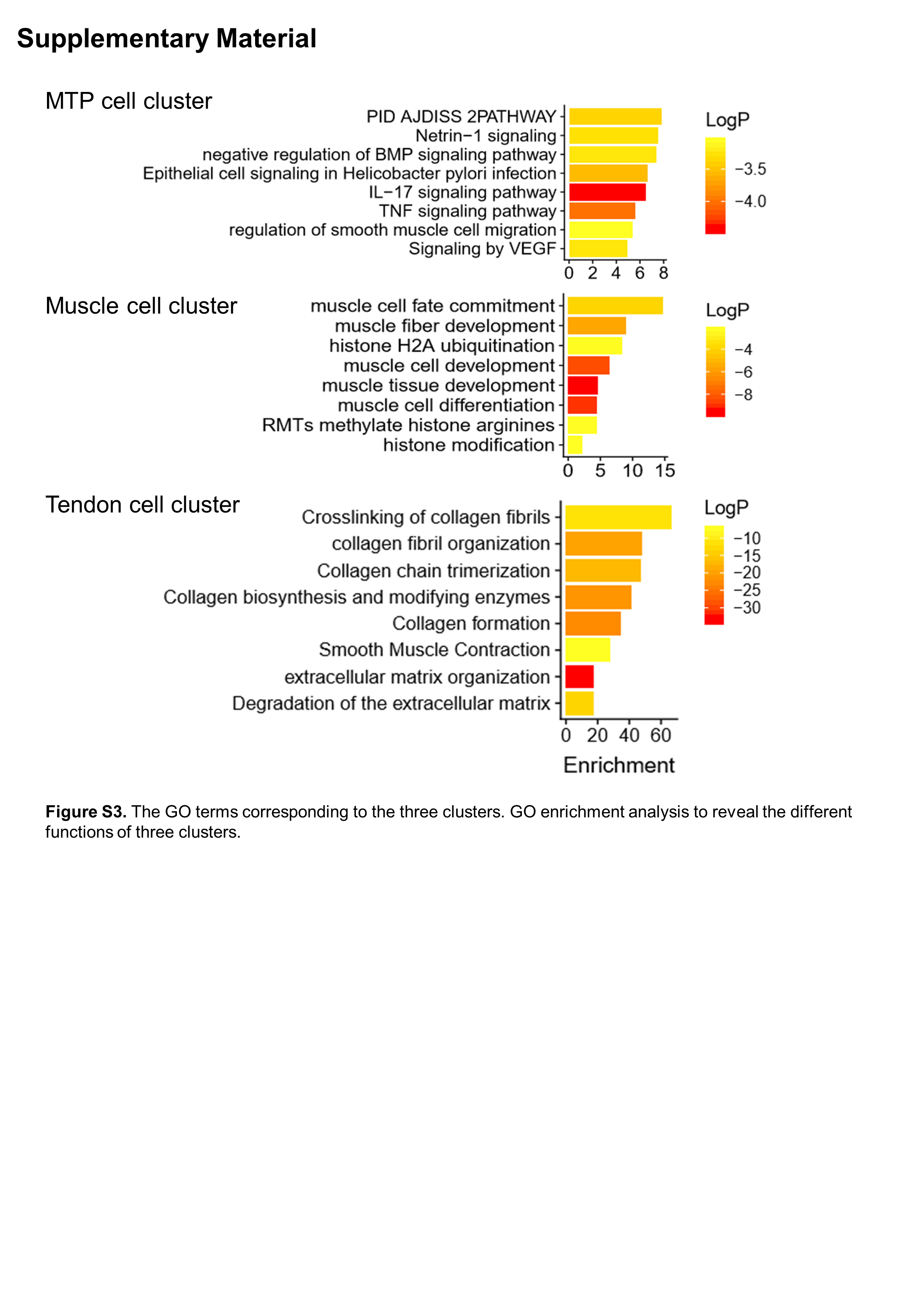


**Figure S3.** The GO terms corresponding to the three clusters. GO enrichment analysis to reveal the different functions of three clusters.


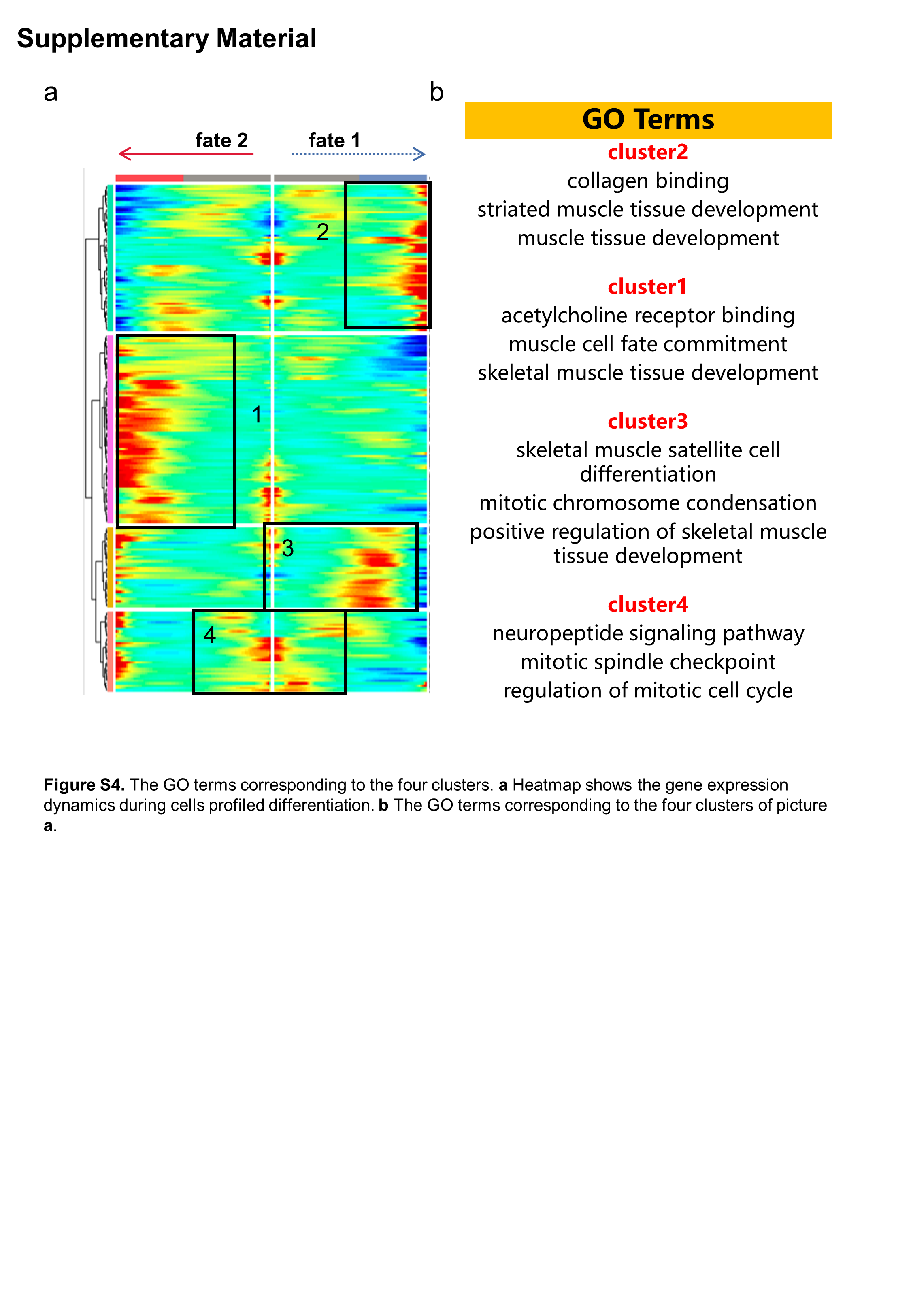


**Figure S4.** The GO terms corresponding to the four clusters. **a** Heatmap shows the gene expression dynamics during cells profiled differentiation. **b** The GO terms corresponding to the four clusters of picture **a**.


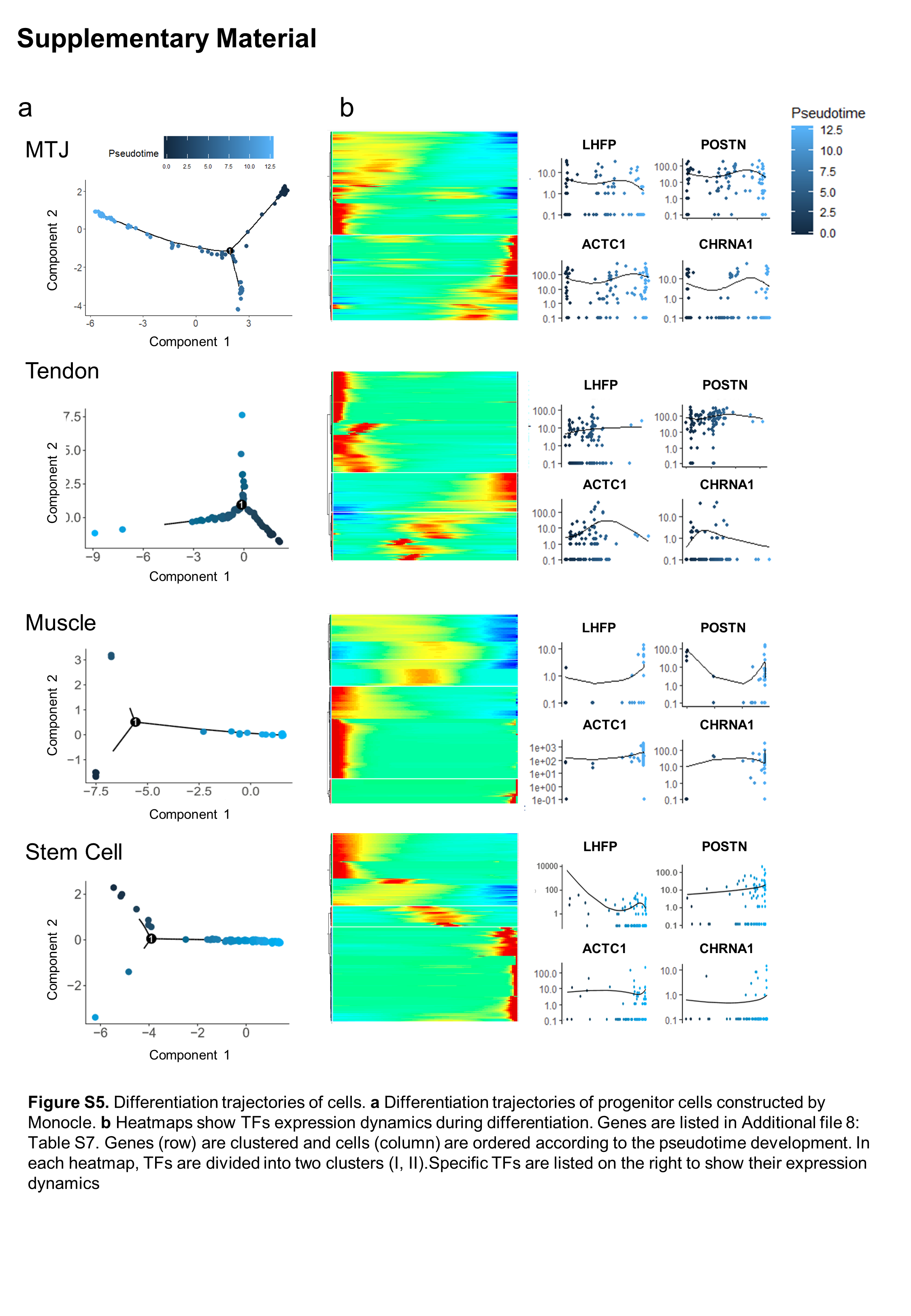


**Figure S5.** Differentiation trajectories of cells. **a** Differentiation trajectories of progenitor cells constructed by Monocle. **b** Heatmaps show TFs expression dynamics during differentiation. Genes are listed in Additional file 8: Table S7. Genes (row) are clustered and cells (column) are ordered according to the pseudotime development. In each heatmap, TFs are divided into two clusters (I, II).Specific TFs are listed on the right to show their expression dynamics


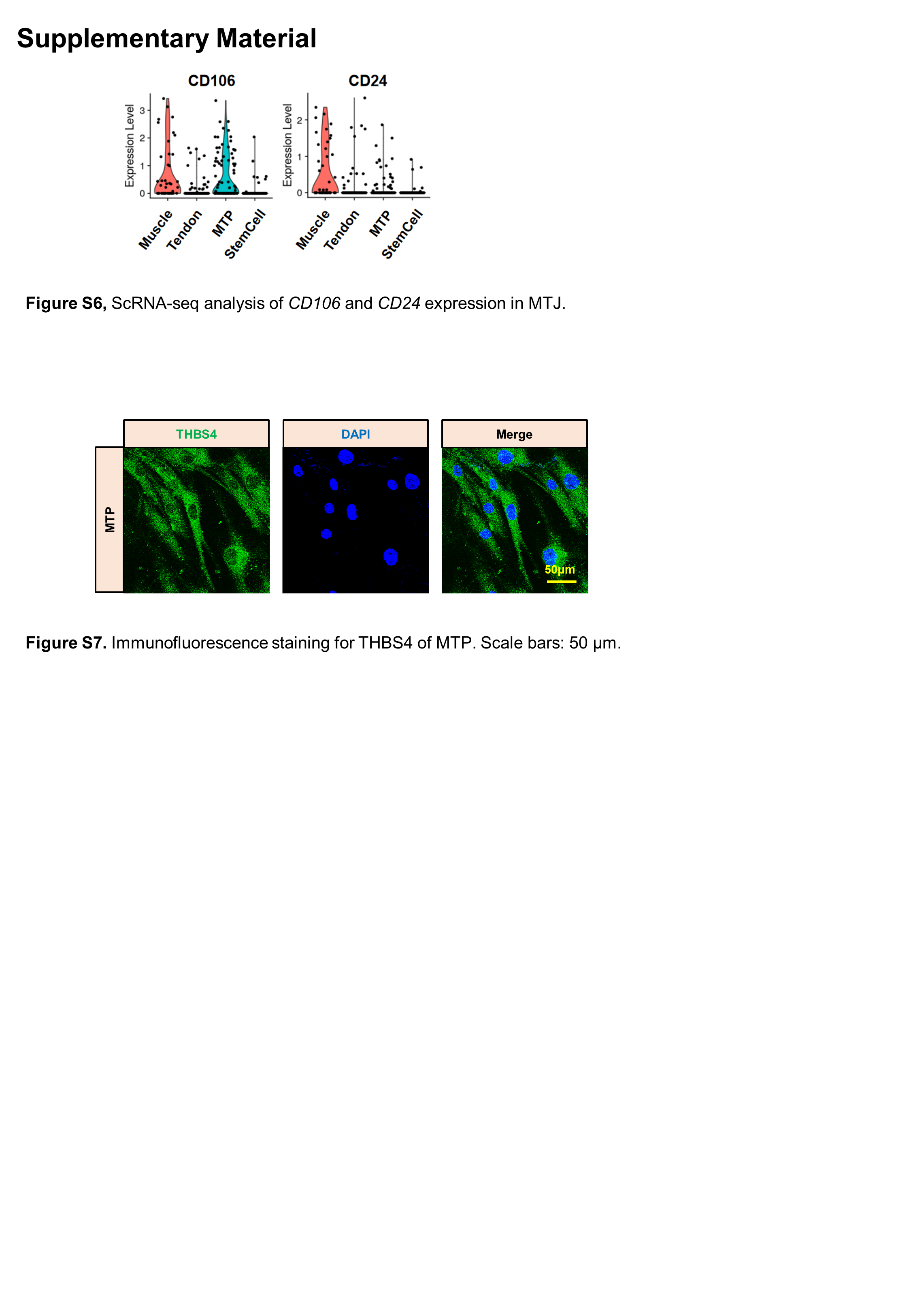


**Figure S6.** ScRNA-seq analysis of CD106 and CD24 expression in MTJ.


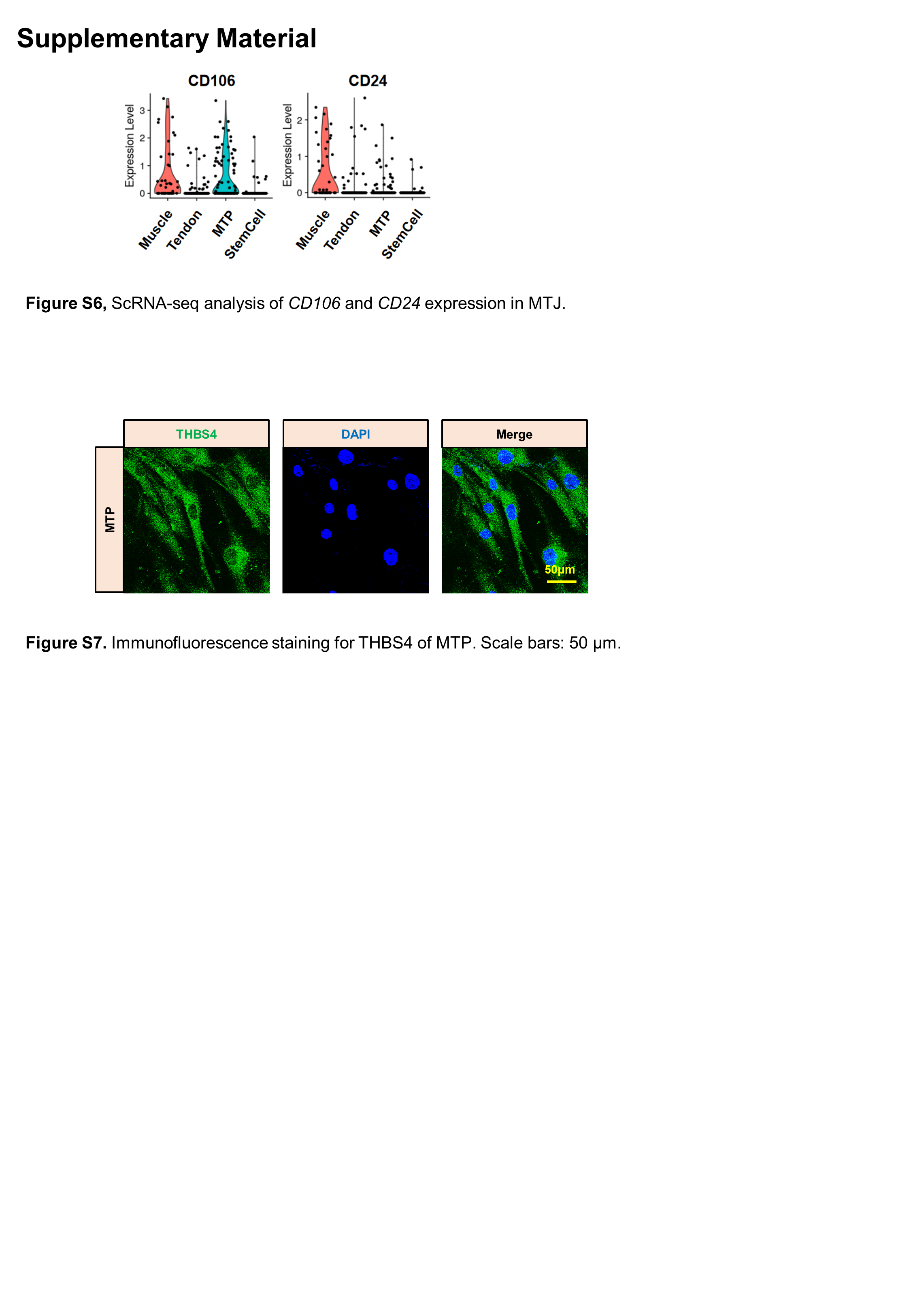


**Figure S7.** Immunofluorescence staining for THBS4 of MTP. Scale bars: 50 μm.


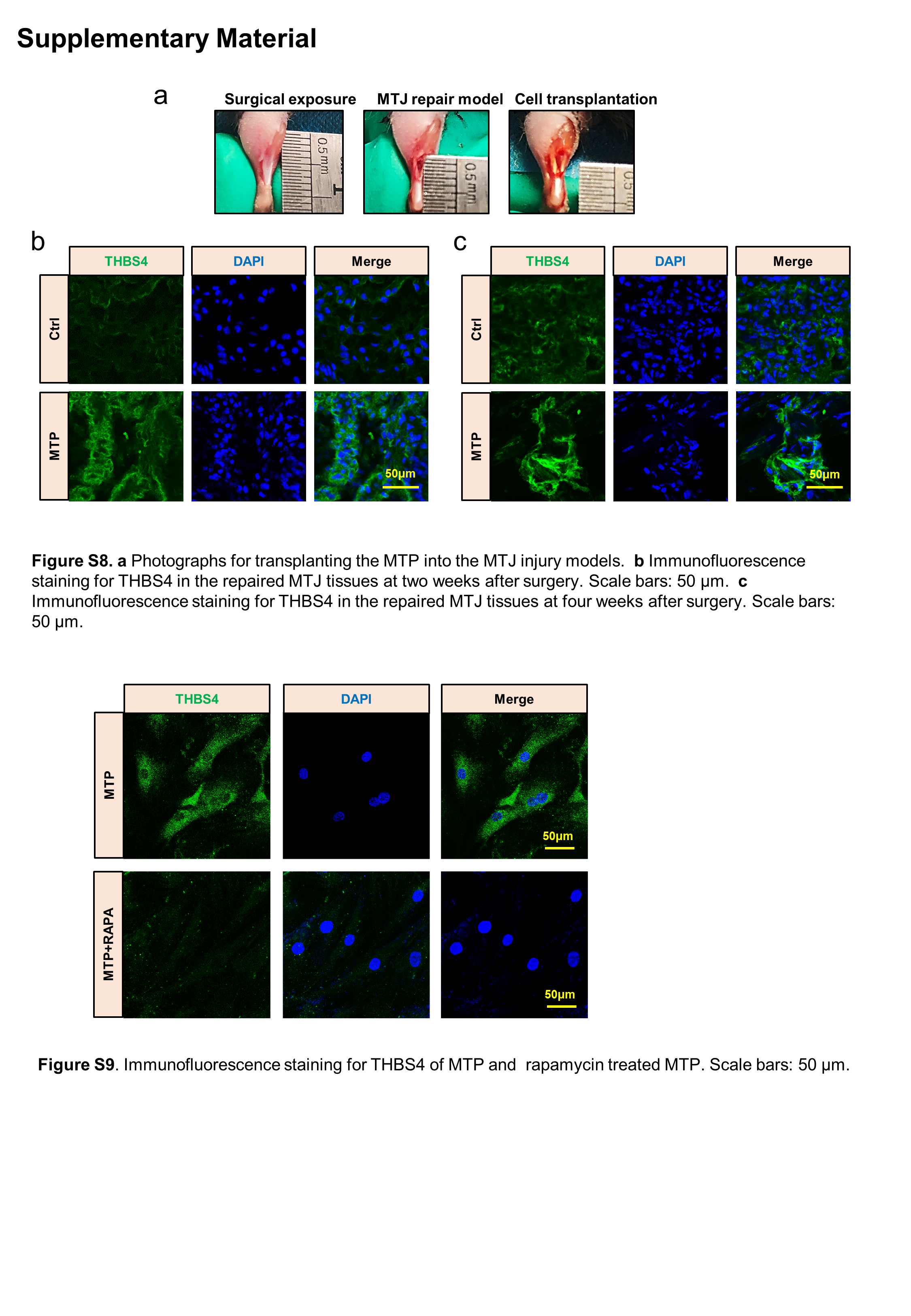


**Figure S8. a** Photographs for transplanting the MTP into the MTJ injury models. **b** Immunofluorescence staining for THBS4 in the repaired MTJ tissues at two weeks after surgery. Scale bars: 50 μm. **c** Immunofluorescence staining for THBS4 in the repaired MTJ tissues at four weeks after surgery. Scale bars: 50 μm.

.


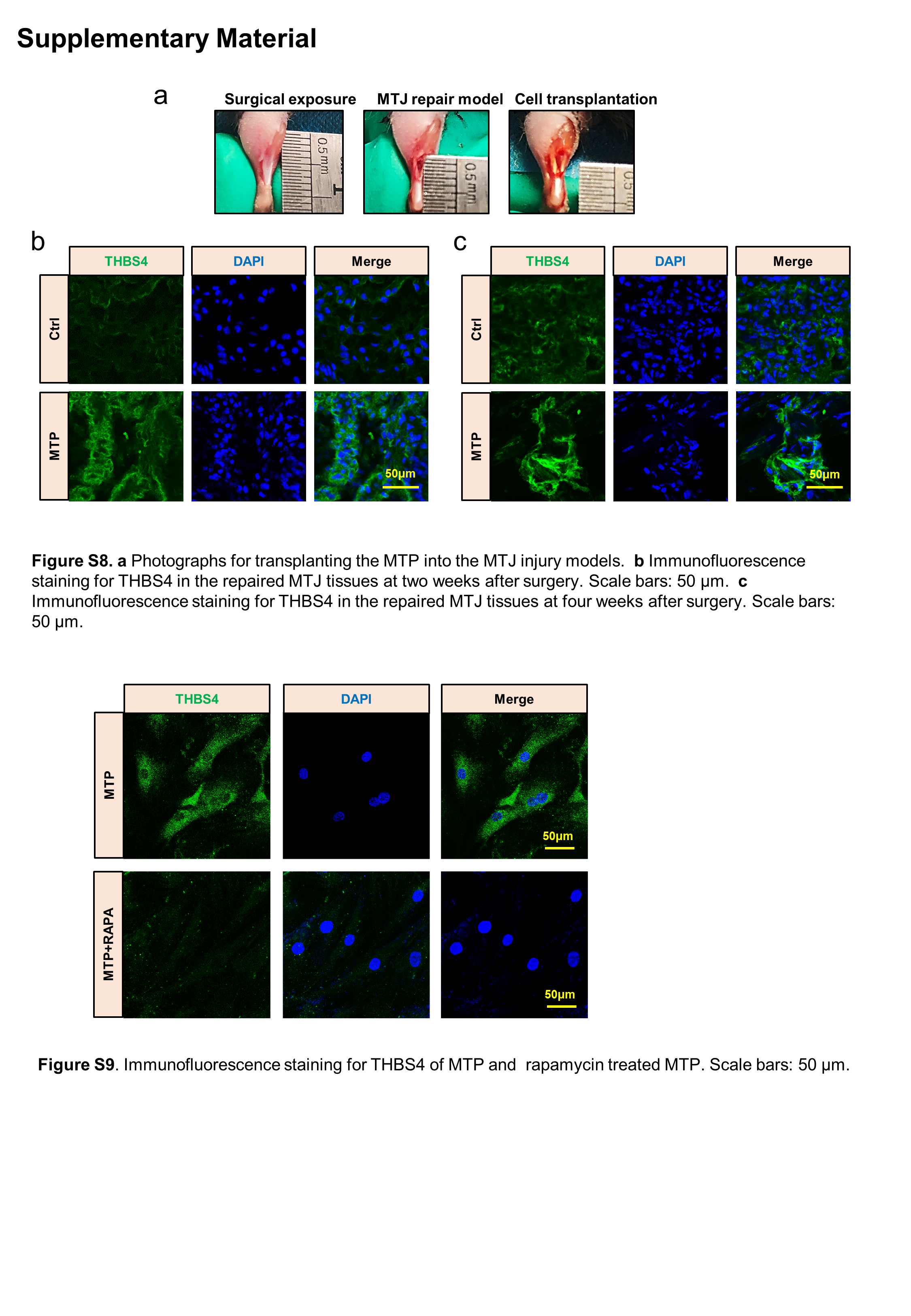


**Figure S9.** Immunofluorescence staining for THBS4 of MTP and rapamycin treated MTP. Scale bars: 50 μm.


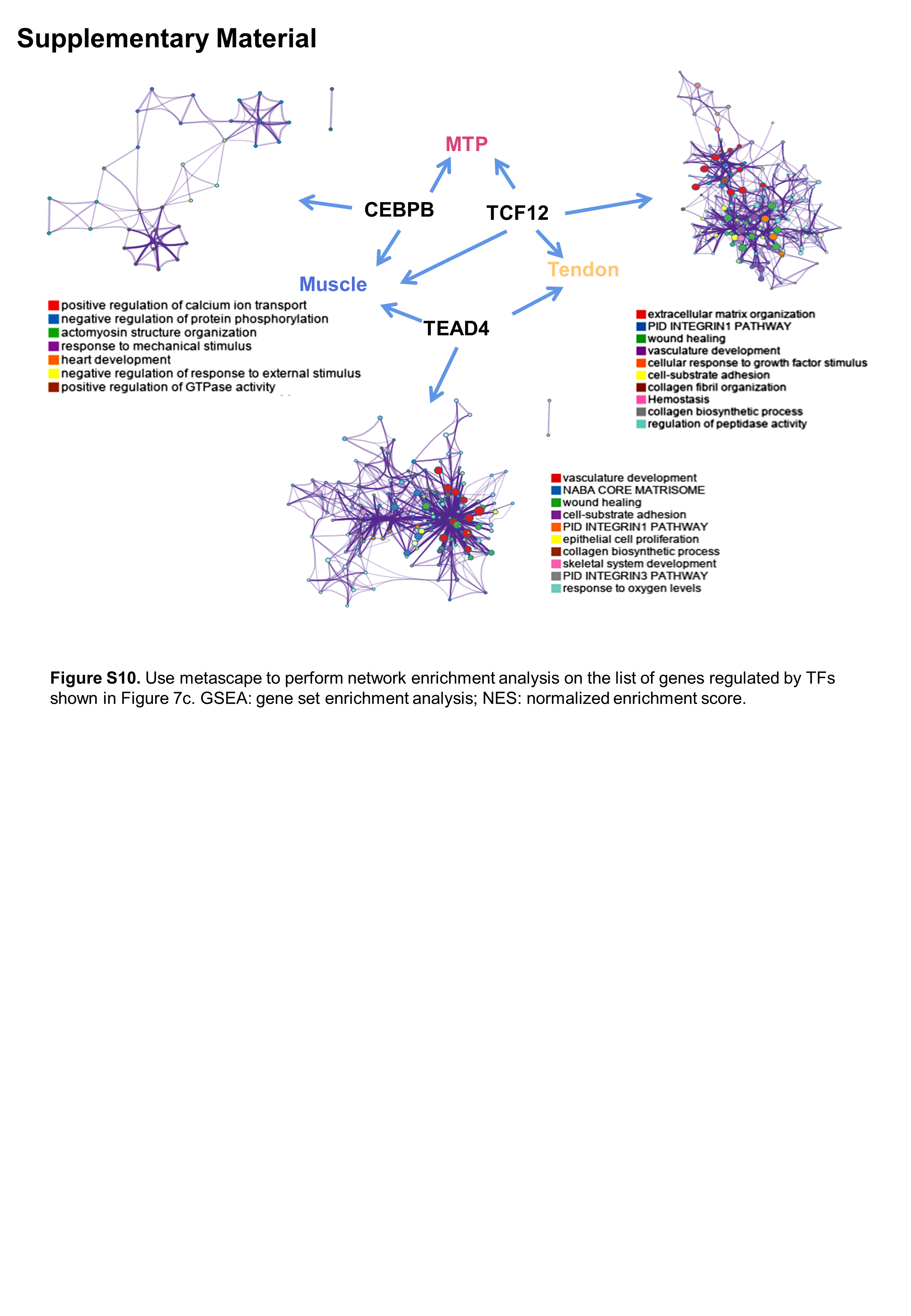


**Figure S10.** Use metascape to perform network enrichment analysis on the list of genes regulated by TFs shown in Figure 7c. GSEA: gene set enrichment analysis; NES: normalized enrichment score.


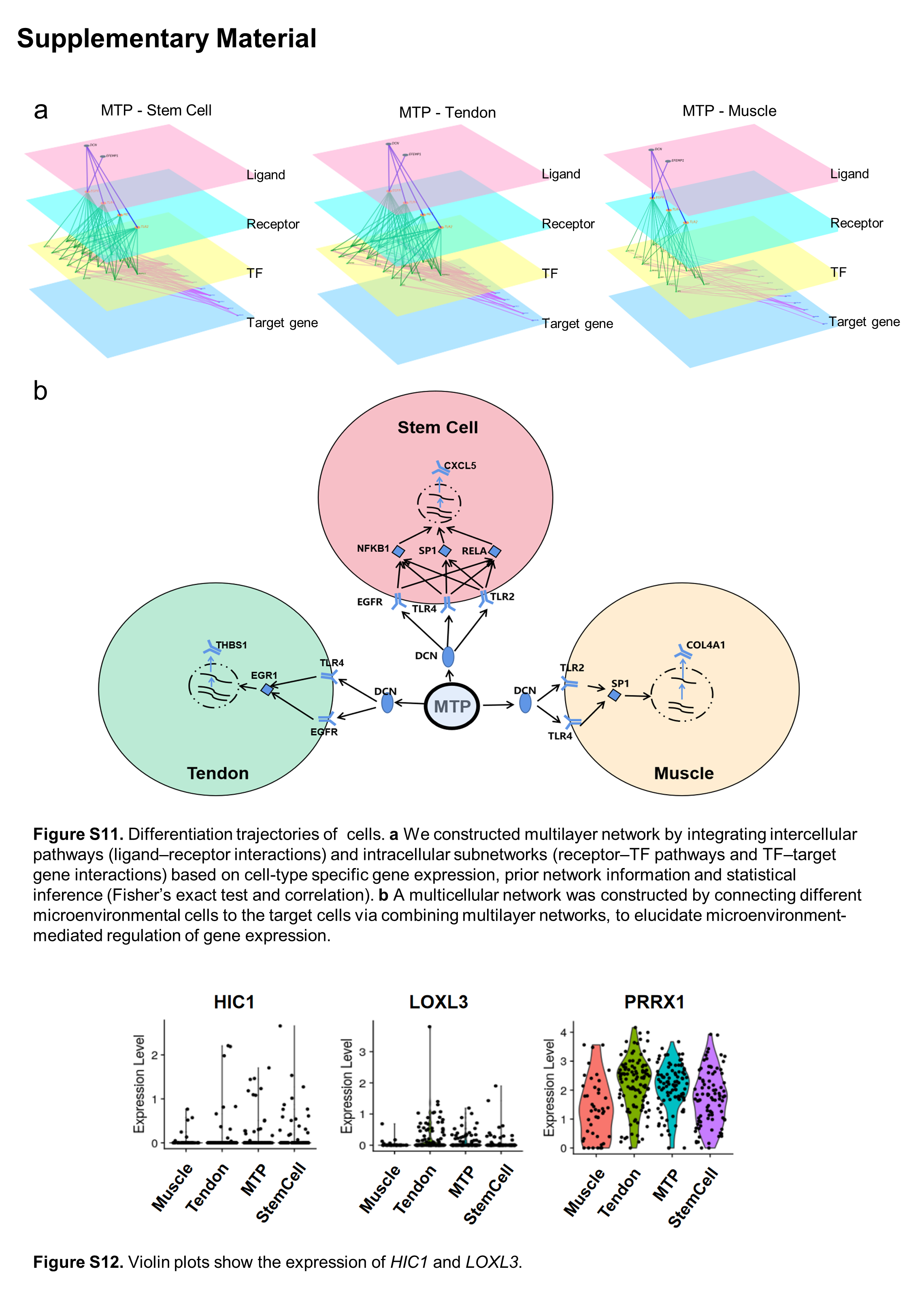


**Figure S11.** Differentiation trajectories of cells. **a** We constructed multilayer network by integrating intercellular pathways (ligand - receptor interactions) and intracellular subnetworks (receptor - TF pathways and TF - target gene interactions) based on cell-type specific gene expression, prior network information and statistical inference (Fisher’s exact test and correlation). **b** A multicellular network was constructed by connecting different microenvironmental cells to the target cells via combining multilayer networks, to elucidate microenvironment-mediated regulation of gene expression.


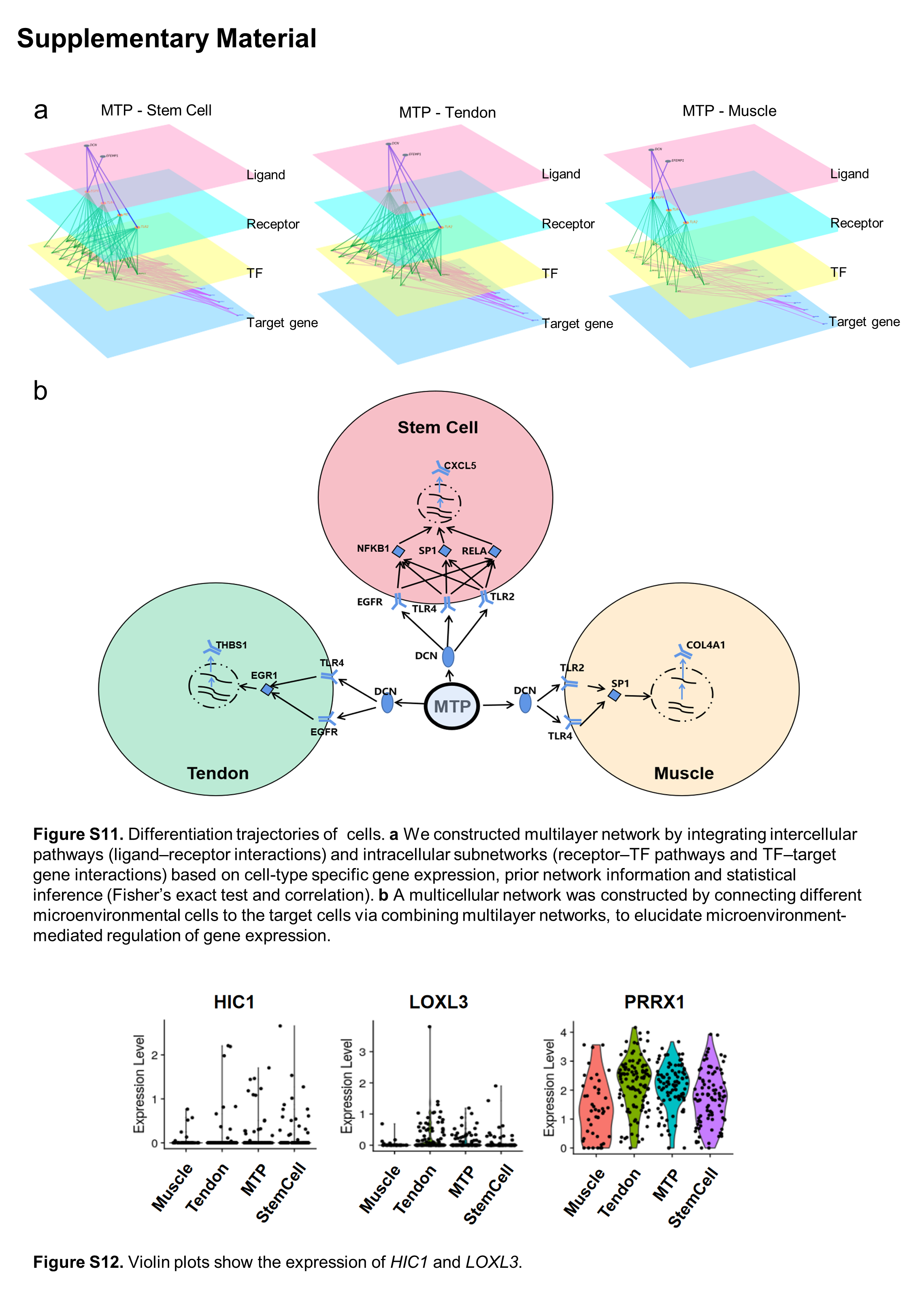


**Figure S12.** Violin plots show the expression of *HIC1*, *LOXL3* and *PRRX1*.

**Methods**

**Tissue Sampling**

All procedures and protocols for humnan samples were conducted with the informed consent and approval of the Ethics Committee of the Second Affiliated Hospital, School of Medicine, Zhejiang University(#16zju20160271). All animal samples had ethical approval from the Institutional Animal Care and Use Committee of Zhejiang University (#zju20190049).

**Single-Cell Capture, cDNA Library Preparation and Sequencing**

We used Fluidigm C1 system and C1 high-throughput integrated fluidics circuits (HT IFCs) to perform the single-cells capture and library construction. cells at 5 × 105 cells/ml were loaded onto two 17–25 μm C1 Single-Cell Auto Prep integrated fluidic circuits (Fluidigm) and cell capture was performed according to the Fluidigm protocol (PN 1009886). Imaged by phase-contrast microscopy to assess the number of cells per capture site. Empty capture wells and wells containing multiple cells or cell debris were discarded for quality control. The cDNA reaction products were quantified using the Qubit™ dsDNA HS Assay Kit. Multiplex sequencing libraries were generated using the TruePrepTM DNA Library Prep Kit V2 and the Nextera XT Index Kit (Illumina). Libraries were pooled and subjected to sequencing on an Illumina HiSeq 2000 (Illumina). We used Illumina (TaKaRa) to prepare cDNA libraries for Illumina sequencing. The diluted cDNA reaction products were converted into mRNA-seq libraries using the Nextera XT DNA sample preparation kit (Illumina, FC-131-1096, -2001 and -2002) following the manufacturer's instructions.

**Histology and Immunofluorescence**

The harvested specimens were immediately fixed in 4% (w/v) paraformaldehyde in phosphate-buffered saline for 24 h (n = 3). The samples were then dehydrated through an alcohol gradient, cleared, and embedded in paraffin blocks. Histological sections (7 mm) were prepared using a microtome and subsequently stained with hematoxylin and eosin (H&E), and Safranin O.

For immunofluorescence, the following antibodies were used: DAPI(Beyotime, C1002), COL14A(Abcam, ab5808), THBS4(Abcam, ab176116), ASB5(Abcam, ab151452) and T1/TNT(Abcam, ab155028); secondary antibodies conjugated with Alexa Fluor 488 or Alexa Fluor 546 or Alexa Fluor 647 or Alexa Fluor 594 fluorescent dyes (Thermo Fisher Scientific) were used for immunofluorescent staining. The stained specimens were photographed digitally under confocal microscope (Nikon A1R).

**Processing of the scRNA-Seq Data**

Raw sequencing reads was processed with Perl scripts to ensure the quality of data used in further analysis. For quality control, The authors excluded cells in which less than 2000 genes or more than 8000 genes were detected and genes that are detected in less than 10 cells.

After obtaining the digital gene expression data matrix, The we used Seurat (2.4.3) for dimension reduction, clustering, and differential gene expression analysis[31] in R (3.5.0). The specific steps are as follows:

1) Input data

We prepared the muscle-tendon junction cells as single-cell samples. After high-throughput single-cell sequencing and basic data processing, we obtained the scRNA data set. The data set consists of 546 cells and expresses 14979 genes in total. Import the data into Seurat, perform QC, standardization, noise reduction, and reduce the impact of batch differences in steps, perform PCA analysis, and obtain PCHeatmap after visualization, and observe the differences in gene expression on each PC.

2) Select the number of principal components

Use the JackStrawPlot() function to draw the rolling stone graph and select the appropriate number of principal components combined with the results of the PCHeatmap graph.

3) Clustering

For clustering, we generate clustering results by setting appropriate resolution parameters in the FindAllCluster() function, and draw tSNE graphs.

4) Looking for two clusters of marker genes

Run the FindAllMarkers() function in seurat to use the default Wlicoxon rank sum test to find marker genes in each cluster.

5) Drawing to observe the differential gene

For each group of Marker genes, use the VlnPlot() function to draw a violin graph, and use the FeaturePlot() function to draw a scatter plot of gene expression to better observe the expression of Marker genes on each cell. Use the Heatmap function to draw a heat map and observe the specific differences of each group.

Then we used Metascape (http://metascape.org/) to perform Gene Ontology analysis (GO) on the respective Marker genes of each group, get the enrichment results, and compare which subgroups are mainly differentially expressed in functions. We ran StemID (https://github.com/dgrun/StemID) to get a histogram of differentiation potential scores. The digital gene expression matrix standardized by CPM (count-per-million) and the specific clustering information obtained after Seurat analysis are used as the input of monocle (monocle 2). Then, the marker genes of the four cell subgroups were used as differential genes, and monocle analysis was performed to obtain the differentiation trajectories of the four subgroups on the pseudo-time series. Connective map method was used to get the interaction between the four subgroups. GSEA was used for GO analysis and signaling pathway analysis. Taking the TFs predicted by the four groups of top100 marker genes on cytoscape(3.8.0) as input, draw a Sankey diagram to show the activity regulation of key stem cell clusters, MTP cell clusters, muscle cell clusters and tendon cell clusters labeled by TFs.
